# A Dual In-Person and Remote Assessment Approach to Developing Digital End Points Relevant to Autism and Co-Occurring Conditions: Protocol for a Multisite Observational Study

**DOI:** 10.2196/71145

**Published:** 2025-10-03

**Authors:** Isabel Yorke, Charlotte A Boatman, Akash Roy Choudhury, Bethany Oakley, Pauline Conde, Heet Sankesara, Yatharth Ranjan, Zulqarnain Rashid, Judith Dineley, Johnny Downs, Christopher H Chatham, Nicholas Cummins, Amos Folarin, Eva Loth, Jan Buitelaar, Declan Murphy, Richard Dobson, Emily Simonoff

**Affiliations:** 1 Department of Child and Adolescent Psychiatry Institute of Psychiatry, Psychology and Neuroscience King's College London London United Kingdom; 2 CAMHS Digital Lab, Department of Child and Adolescent Psychiatry Institute of Psychiatry, Psychology & Neuroscience King's College London London United Kingdom; 3 Department of Forensic and Neurodevelopmental Sciences Institute of Psychiatry, Psychology, and Neuroscience King's College London London United Kingdom; 4 Department of Biostatistics and Health Informatics Institute of Psychiatry, Psychology, and Neuroscience King's College London London United Kingdom; 5 Current Affiliation: Biomarker Research, Shionogi, Inc Florham Park, NJ United States; 6 Pharma Research & Early Development F. Hoffmann La Roche, Ltd Little Falls, NJ United States; 7 Institute of Health Informatics University College London London United Kingdom; 8 NIHR Biomedical Research Center South London and Maudsley NHS Foundation Trust London United Kingdom; 9 Health Data Research UK University College London London United Kingdom; 10 NIHR Biomedical Research Center University College London Hospitals NHS Foundation Trust London United Kingdom; 11 Department of Cognitive Neuroscience Donders Institute for Brain, Cognition and Behaviour Radboud University Medical Center Nijmegen The Netherlands

**Keywords:** autism, mental health, sleep, digital end points, remote measurement technology, wearables

## Abstract

**Background:**

Research priorities for autistic people include developing effective interventions for the numerous challenges affecting their daily living, for example, mental health problems, sleep difficulties, and social well-being. However, clinical research progress is limited by a lack of validated objective measures that represent target outcomes for improvement. Digital technologies, including wearable devices and smartphone apps, provide opportunities to develop novel measures that may reflect everyday experience and complement key clinical assessments. However, little is known about the acceptability and feasibility of implementing digital data collection in this population.

**Objective:**

The primary objective of this study is to evaluate the usability, acceptability, adherence, and feasibility of a dual in-person and remote (ie, at-home) protocol. Secondarily, we aim to explore the properties of certain resulting data with a view to developing novel digital end points for key target outcomes, including social communication, sleep, and mental health.

**Methods:**

Eligible autistic and nonautistic in the AIMS Longitudinal European Autism Project were invited to participate in a digitally augmented in-person Autism Diagnostic Observation Schedule-2 (ADOS-2) and a 28-day remote measurement (RM) protocol involving wearing a Fitbit device, downloading a passive smartphone data collection app, and using 2 active reporting apps.

**Results:**

The first AIMS Longitudinal European Autism Project study participants were enrolled in September 2021 (in-person component) and March 2022 (RM component). To date, 190 participants have taken part in the digitally augmented ADOS-2 component, and 86 participants have been enrolled for the RM protocol. Recruitment is now complete with some RM data collection ongoing until August 2025. Data analysis has commenced, including qualitative framework analysis of feedback interview data coproduced with autism community members, exploration of acceptability and feasibility metrics, pipeline development for ADOS-2 speech analysis, and RM sleep measures.

**Conclusions:**

This study lays important groundwork in understanding the acceptability and feasibility of in-person and remotely implemented digital measurement procedures to capture meaningful outcomes in domains important to improving everyday life for autistic people.

**International Registered Report Identifier (IRRID):**

DERR1-10.2196/71145

## Introduction

### Background

Autism is a lifelong neurodevelopmental condition characterized by social communication differences, restricted or repetitive behaviors and interests, and differences in sensory experience [1]. Presentation across individuals is diverse and often complicated by co-occurring conditions, such as intellectual disability, attention-deficit/hyperactivity disorder (ADHD), mental health problems, epilepsy, and sleep difficulties [2]. Most autistic people face considerable challenges affecting numerous domains of everyday life, such as accessing health care, community activities, education, and employment [3]. Across studies consulting the autism community, major research priorities include enabling activities of daily living and enhancing quality of life by treating co-occurring conditions and alleviating difficulties associated with core characteristics (eg, providing training for social, vocational, and life skills and by making environments more autism friendly) [4]. However, progress in addressing these priorities is limited not only by the development of effective intervention and support strategies but also by the measurement tools available to capture desired changes in response to intervention [5-7].

### Current Measurement

Self- or informant-report instruments and clinical observations or assessments form most outcome measures used in clinical autism research in domains such as social functioning [8], mental health [6], and ADHD [9]. Self-report measures are well-placed to capture real-world experiences and behavior. However, high rates of alexithymia and difficulties or differences in interpreting questionnaire items may present specific challenges for autistic people reporting on their own feelings and behaviors [10,11]. Parents, teachers, and other informants reporting on behaviors in different contexts share only modest variance, with characteristics such as parenting stress accounting for discrepancies [12,13]. When evaluating psychosocial treatment approaches, which play a key role in supporting autistic people and their families, findings may be impacted by difficulty in blinding informants involved in the treatment itself [14]. Finally, even where blinding is possible, self- or informant-report measures may show spontaneous improvement in the absence of treatment, which makes treatment effects more difficult to establish [15,16].

Assessments carried out by clinicians and researchers mitigate certain limitations; for example, in pharmacological trials targeting core autistic features (eg, social communication difficulties), placebo effects were lower for clinician versus caregiver ratings [16]. However, these are time-consuming and therefore costly to implement at scale. They may also be unrepresentative of everyday behavior or experiences because they are often conducted in unfamiliar settings that are potentially anxiety provoking and challenging for autistic people to access. Finally, whether self- or informant reported, such measures are often ordinal and limited in their effective resolution, compromising sensitivity [2] and inflating thresholds for detecting clinically meaningful results [17]. Given the profile of strengths and limitations of measurement options based on human judgment, many authors have called for the development of objective measurement tools to complement these.

### Digital Measurement Opportunities

Table 1 summarizes potential areas in which digital technology could address the limitations of current measurement, as well as outlining potential challenges for measure development based on these, including those that may be particularly problematic in autism research. Smartphones and wearable devices, such as smartwatches, are widely used by the general population and are subject to commercial optimization; thus, their application in health research may benefit from already honed acceptability. They offer an array of passively collected data types (ie, not requiring user input), including physiological (eg, heart rate), lifestyle (eg, sleep and activity levels), and nearby environmental data (eg, light levels). These may provide more objective data indexing characteristics and symptoms of interest while reducing challenges associated with subjective reporting. Furthermore, their unobtrusiveness may reduce Hawthorne effects (ie, behavioral change due to being observed) [18]. Smartphones also provide the opportunity to collect active measures efficiently and repeatedly in everyday life (eg, collecting mood data via ecological momentary assessment) [19]. Although subjective, these data have high ecological validity and minimize recall effects. They provide context for objective data and enable investigation of fluctuation in subjective symptom levels. At the same time, modern analytical methods, such as machine learning, provide tools for efficiently extracting and identifying meaningful patterns in large datasets. Suitably large datasets are readily produced by wearables, as well as through video, audio, and natural language recording, and can now be processed much more efficiently than via traditional approaches such as manual coding. Together, such technology may enable efficient measurement with relatively low burden to participants and high ecological validity. Such characteristics hold particular promises for clinical research, in which symptom changes would ideally be tracked over time, and early detection of a subtle change may be valuable.

**Table 1:** Summary of current measurement limitations, opportunities for digital technology to address these, and potential challenges therein.

Domain	Limitation of current measurement	Opportunity for digital technology	Potential challenges
Meaningful capture of real-world outcomes	Difficulties associated with self-reporting and recall effects Observations and assessments are often conducted at discrete time points and in unfamiliar situations	Reporting in the moment via smartphone may alleviate difficulty remembering thoughts, feelings, and behavior over weeks or months Real-world, real-time data collection offers potential for high ecological validity	Limits inclusion of certain groups, for example, those without a smartphone and those who find technology less accessible or trustworthy. Daily reporting for long periods may be burdensome. Both these points may disproportionately affect autistic individuals.
Measurement error and bias	Susceptibility to attention or placebo effects; difficulty blinding informants in psychosocial intervention research	Objective data capture possible via passive physiological and environmental sensors	Subjectivity may still be present, for example, in analytic choices and model training. Models relying on neurotypical data may misclassify data from autistic individuals.
Scalability	Observations and assessments by researchers or clinicians are expensive and time-consuming Repeated and lengthy questionnaire administration may become burdensome	Potentially highly scalable across individuals and over time Passive data collection could reduce the burden of repeated or long-term data collection	Wide variation in affordability of devices; many devices are inaccessible in lower-resource research settings. Wearable device comfort and data privacy concerns associated with passive data collection may affect adherence and exclude certain groups, for example, those with sensory sensitivities.
Capture of differences	Difficult for humans to detect subtle differences across or within people	Digital sensors can capture fine-grained differences	Processing and interpretation of large amounts of data is challenging, has an environmental impact, and often lacks validation against well-understood instruments.

For these reasons, digital measurement has drawn interest in diverse physical health applications, such as wearable sensors for monitoring recovery in oncology patients and patients who have had stroke [20,21], monitoring disease progression in Parkinson disease [22], and identifying epileptic seizures noninvasively [23]. Elsewhere, smartphone apps and wearable wristbands have been explored for use in predicting depression relapses [24,25]. In autism research specifically, several digital measurement systems have been developed. These include in-person autism diagnostics or brief conversation-based assessments using machine learning to quantify elements of social communication from videotaped interactions [26-29]. Similar techniques have also been applied remotely, including at-home attention and emotion analysis based on computer vision [30]. In the domain of sleep, objective monitoring via actigraphy has been applied relatively widely [31-33]. Integrating laboratory-based and wearable biosensors with a web-based and mobile app to collect objective and caregiver-report data, the Janssen Autism Knowledge Engine (JAKE) system [34] has been fruitful in exploring existing and novel measures relating to autism and co-occurring symptoms in the domains of sleep [35], prediction of diagnostic groups from multimodal biosensor data [36], and daily caregiver-reported mood and behavior [37]. Published research using the JAKE system has focused on a sample of caregivers and their autistic or nonautistic children, who are predominantly children or young adults of average intellectual ability. However, to our knowledge, a comprehensive system such as the JAKE system has not been developed to capture self-report and passive smartphone data alongside wearable and in-person assessment among autistic adolescent and adult smartphone users.

Furthermore, despite growing research interest, very little has been published on the acceptability, feasibility, and implementation challenges of digital assessments in autism (although important work on evidence-based practice for developing digital supports for autistic people has been published [38]). For the JAKE system, caregiver feedback was sought and showed encouraging findings in terms of usability and device comfort; however, the experiences of the autistic participants themselves were not directly captured. Autistic people's experiences of research supported by digital technology are likely to differ in several ways from those of other populations, as is the analysis and interpretation of the data yielded. First, sensory sensitivities may affect the comfort of wearable devices. This means that important data may be missing, for example, if people find a wristband uncomfortable to wear at night in a sleep study [39]. On the other hand, wristbands may be more widely tolerated than scalp electrodes such as those used in polysomnography [40] and innovation in the characterization of sleep from data acquired via wristbands (eg, actigraphy and pulse rate monitoring) may enable the inclusion of a wider proportion of the autistic population. Second, higher rates of intellectual disability may affect the usability of smartphone apps, and differences in communication and emotional literacy may affect the way in which objective data correspond to reported subjective experience. Third, analytical tools and pipelines developed in other populations may function differently in the autistic population. Care must be taken to anticipate potential neurotypical biases in the ways that features of speech, social interaction, and facial expressions are classified in machine learning tools trained in other populations.

### Study Approaches and Target Domains

**Figure 1:**
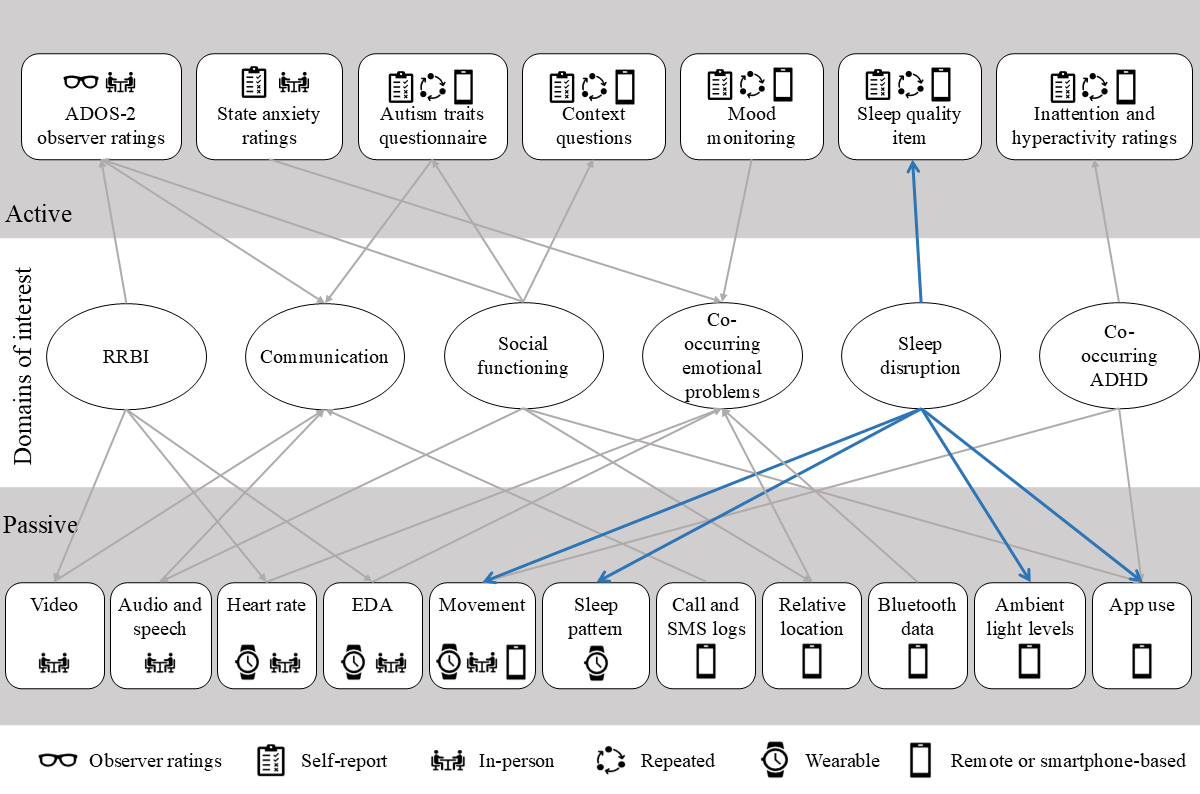
Conceptual depiction of study approaches to exploring individual and combined potential for candidate data collection modes to index variance in domains of interest. Arrows represent potential indexing of domains of interest by data collection modes. For example, sleep disruption (blue arrows) may be indicated by a self-report sleep quality rating and also by several passive data sources. ADHD: attention-deficit/hyperactivity disorder; ADOS-2: Autism Diagnostic Observation Schedule-2; EDA: electrodermal activity; RRBI: restricted or repetitive behaviors and interests.

This study protocol comprises a dual in-person and remote approach to digital measure development in the domains of autistic characteristics (social communication and restricted or repetitive behaviors and interests) and selected co-occurring difficulties (mood, sleep, inattention, and hyperactivity). Our primary focus is on evaluating the acceptability and feasibility of the methods developed. Co-occurring domains were selected based on being (1) associated with everyday challenges, (2) highlighted by autistic people as important targets for improvement, and (3) putatively amenable to digital measurement. As depicted in Figure 1, we aim to enable the evaluation of a range of candidate data collection methods for their individual and combined potential in indexing these target domains.

Our in-person approach explores digital augmentation to the Autism Diagnostic Observation Schedule-2 (ADOS-2) [41], a widely used in-person observational assessment, a gold-standard instrument in informing autism diagnosis. The ADOS-2 has been used in clinical studies to evaluate intervention effects on social communication [42-44]. Its scoring system is designed to allow human administrators to differentiate reliably between several levels of intensity across a range of behaviors characteristic of autism. Because fine-grained discriminations are challenging for humans, this system may not be ideal for identifying subtle changes, for example, early response to an intervention [45]. Our digital augmentations to the ADOS-2 comprise video recording from 3 angles, audio recording for speech transcription, and a wearable wristband recording heart rate using photoplethysmography, electrodermal activity (EDA), and accelerometer data. These digital augmentations are intended to enable exploration of objective data to complement subjective observer ratings of social communication and repetitive and restricted behaviors and of the underlying autonomic demands of this assessment.

Existing research has shown autonomic correlates of outcomes of interest within the autistic population, such as social function [46], anxiety [47], and depression [48]. Such outcomes are among those that are both key sources of everyday distress and difficult to verbalize for many autistic people. Thus, there is an interest in discovering objective autonomic markers that may underlie states of subjective distress and may be sensitive to treatment response. The ADOS-2 provides a standardized series of presses mimicking everyday interpersonal interactions that present various challenges to autistic people. Therefore, it is well-placed for studying autonomic responses during such interactions.

We will also use a 28-day remote measurement (RM) period by using a commercial wearable device and both passive and active smartphone-based measures. Passive smartphone data collection will use an app that records data continuously from smartphone sensors (eg, motion sensors, GPS location, and Bluetooth connectivity) and logs (eg, app use and call and SMS logs). Such apps have contributed elsewhere to assessing mental health [49,50], and diverse aspects of social interaction and environment [51-53]; however, they have not been widely applied in autism. In this study, both active and passive approaches seek to understand trade-offs between data quality, acceptability to participants, and feasibility of implementation.

For both in-person and remote assessments, we developed our approach in a stepwise manner. First, autism community representatives were consulted to discuss potential opportunities and pitfalls for using candidate technologies in autistic populations, to select specific devices, to maximize procedural acceptability, and to identify potential facilitators and barriers for participation. Protocols were then piloted before implementation as part of the third time point of the AIMS Longitudinal European Autism Project (LEAP) [54,55]. As a large, well-characterized cohort comprising both autistic individuals and comparison individuals, data collection in LEAP enables a detailed understanding of participant characteristics that may affect acceptability and feasibility and how our measures relate to existing gold-standard and widely used conventional measures.

### Study Objectives

The primary aim of this study is to evaluate, via quantitative and qualitative methods, the acceptability and feasibility of the measurement systems developed. Primary acceptability outcomes are uptake, adherence, completion rates for each component, and qualitative feedback on participant experience for RM. Primary feasibility outcomes are data availability and proportion of usable data relevant to each target domain. Secondarily, we aim to develop measures from the resulting data relevant to core autism characteristics (eg, social communication) and co-occurring conditions (eg, mental health and sleep problems). Finally, we aim to explore the resulting measures in terms of the association between subjective and objective measures and psychometric properties. In so doing, we hope to identify key candidate measures that show promising characteristics for further validation. This paper will describe the overall design, protocols for in-person and remote assessment, and key target analyses before discussing the potential impact, strengths, and limitations of our approaches.

## Methods

### Overall Design

#### Autism Community Involvement

In developing our protocol, we involved community representatives in a series of online focus groups aiming to explore community views on practical and ethical concerns regarding the use of digital technology in outcome measure development. Community views informed key elements of the protocol, such as study information processes, choice of wearable devices, and the interview schedule for qualitative feedback.

#### Participants

Data collection has been implemented primarily as part of the third (and, at the time of writing, most recent) time point of the LEAP study [54,55]. LEAP constitutes a large, well-characterized cohort recruited from centers across Europe: King’s College London (KCL), University of Cambridge (UCAM), Radboud University Medical Centre, Nijmegen (RUMC), University Medical Centre, Utrecht, and Central Institute of Mental Health, Mannheim. At inception, LEAP included 437 autistic participants and 300 nonautistic participants aged between 6 and 30 years who have undergone repeated comprehensive phenotyping at 3 time points (T1: 2014-2017, T2: 2015-2017, and T3: 2020-2024). This includes gold-standard autism clinical profiling (ie, ADOS-2, Autism Diagnostic Interview-Revised, and Vineland Adaptive Behavior Scales); neurocognitive assessments; co-occurring symptom assessments; brain imaging; biochemical markers; and genomics. Participants with an IQ in the typical range were recruited in a 3:1 male-to-female ratio within age bands defined as (A) adult (aged between 18 and 30 y), (B) adolescent (aged between 12 and 17 y), and (C) child (aged between 6 and 11 y). Participants aged between 12 and 30 years with mild intellectual disability (IQ 50-74) were additionally recruited to a fourth schedule (D). The eligibility criteria for autistic participants were an existing clinical diagnosis according to the *DSM-IV* (*Diagnostic and Statistical Manual of Mental Disorders* [Fourth Edition]); *DSM-5* (*Diagnostic and Statistical Manual of Mental Disorders* [Fifth Edition]); or *ICD-10* (*International Statistical Classification of Diseases, Tenth Revision*) criteria.

Preliminary recruitment figures for LEAP T3 indicate that 225 autistic participants and 132 nonautistic participants took part across sites. A sample of 36 autistic participants and 44 nonautistic participants but with epilepsy (referred to as the LEAP epilepsy sample) was additionally recruited at KCL to complete the LEAP T3 protocol, including digital measures. All autistic participants in schedules A and B (ie, those aged ≥12 years) who were contactable at T3 at the KCL, UCAM, and RUMC sites (including University Medical Centre, Utrecht participants, who were seen at RUMC) were eligible to take part in our digitally augmented ADOS-2. In total, 30 nonautistic participants from across sites and schedules were also to be recruited. For RM, additional eligibility criteria were enrollment at KCL or UCAM (non-UK sites were not included because app translation was beyond our scope), independent use of an up-to-date Android (Google LLC) smartphone (version 8 or later), and willingness to try wearing the Fitbit Inspire 2 device as much as possible (day and night) for 28 days. There were no additional exclusion criteria to not meeting the inclusion criteria. We planned to only include Android users because our passive smartphone data collection app is only compatible with Android smartphones. Although we considered providing Android study phones to participants, we reasoned that these additional smartphones were unlikely to be used in a habitual manner and would thereby compromise ecological validity. Participants indicating interest but also a concern that they would find the Fitbit uncomfortable were invited to try to participate. It was emphasized that they would be free to discontinue wearing the Fitbit at any time or work with researchers to find a more comfortable strap. Finally, participants in schedule D were also eligible for each component, according to the same criteria, where it was felt that the protocol would be manageable within the context of overall demands presented by the wider LEAP T3 protocol and adequately understood for the purpose of providing informed consent. Participants in LEAP epilepsy were also invited to take part in both components according to the same criteria.

In addition to data collection in the LEAP sample, we recruited a volunteer sample of 25 nonautistic individuals or without known neurodevelopmental, neurological, mental health, or sleep conditions (RM comparison sample) to participate in an extended version of the RM component only. While data collection in LEAP and LEAP epilepsy is complete, data collection in our RM comparison sample is ongoing and will be completed in August 2025.

#### RADAR-Base Data Collection Platform

Both in-person and remote assessments use the RADAR-base platform for digital data collection and management [56]. The platform comprises the collection of multiple data sources, for example, passive and active data collection smartphone apps, as well as application programming interface integration for ingesting third-party (eg, Fitbit) data, secure data storage systems, and a management portal for linking data sources to participant identifying codes. The active RM technology (aRMT) app is a questionnaire administration app that allows a customizable schedule of questionnaires to be administered. The use of RADAR-base enables the secure collection and management of automatically time-stamped active and passive data for downstream analysis. The passive RM technology (pRMT) app [23,50] collects smartphone sensor data (eg, movement, relative location, light levels, and app use) as well as data from integrated wearable devices (eg, the Empatica E4 device used in this study).

### In-Person Assessment

#### Procedure

The digitally augmented ADOS-2 is implemented in LEAP T3 as part of a 2-day in-person assessment visit. Cameras and a lapel microphone are set up before the participant enters the room (as depicted in Figure S2 in Multimedia Appendix 1 [27,28,49,57-75]). At the beginning of the assessment, participants are introduced to the Empatica E4 wristband and shown how to put it on their nondominant wrist. Video recording is then started on all cameras, and ceiling lights are switched off and on again as a synchronizing marker. The E4 is then turned on, and connection with the RADAR passive app is automatically established. Before starting the ADOS-2 assessment, participants complete a pencil-and-paper measure of state anxiety (detailed in subsequent sections). The ADOS-2 assessment is then administered according to standard protocol. Data collection started in September 2021, while certain social distancing and enhanced hygiene policies were still in place. We accommodated these via several adaptations to usual ADOS-2 procedures (summarized in Table S1 in Multimedia Appendix 1). The exact implementation for each “ADOS-2 informed assessment” was allowed to differ according to current site-specific policies and guidelines and was documented in case report files. For example, participants and administrators sat at a distance (eg, 2 m), clear masks were used, and certain ADOS-2 materials were adapted or removed. Between each ADOS-2 activity, the participants are asked to wave the wrist on which they were wearing the E4 to create an event marker. At the end of the assessment, recording is stopped, the E4 wristband is removed, and participants fill in the state anxiety measure again. Participants are also invited to wear the E4 wristband during a separate electroencephalography and eye-tracking task battery that forms part of the LEAP T3 visit. This acts as a comparison condition with lower social interaction demands.

#### Measures

##### Empatica E4

The Empatica E4 is a wrist-worn device that captures motion via accelerometer, heart rate via photoplethysmography, and EDA activity via electrodes on the device or via finger attachments (Figure S1 in Multimedia Appendix 1). Previous research has shown poor accuracy for wrist-worn EDA capture [57], and our own piloting demonstrates better capture using finger electrodes. Therefore, we use these unless participants find them uncomfortable, in which case we use the on-device electrodes. This option is provided primarily to maximize collection of photoplethysmography and motion data from the E4 and to explore acceptability of EDA electrode options in this context. This is because EDA readings differ across sites [76], and our protocol is not optimized for wrist placement (to date, all participants willing to wear the E4 were happy with finger placement). E4 data are ingested via the RADAR pRMT app installed on a study-owned compatible Android smartphone. The RADAR pRMT app is configured to pair automatically with the device via Bluetooth to ingest data. The Empatica E4 has been shown elsewhere to be well tolerated in naturalistic observation sessions involving autistic youth and to produce usable data for prediction of overt behavior from physiological data [77].

##### Video and Audio Recording

Video data are captured using commercially available high-definition video cameras, exporting at a minimum of 1920×1080-pixel resolution and 24 frames/second sampling rate, from three angles: (1) recording participant face from researcher perspective, (2) recording researcher face from participant perspective, and (3) recording the scene from observer perspective (camera setup is shown in Figure S2 in Multimedia Appendix 1). Cameras are mounted on tripods to minimize movement. The exact room setup varies across sites and sometimes within sites due to the use of different rooms and the constraints imposed by furniture size and layout. It is also not possible to standardize room acoustics and ambient lighting due to windows and differences in electrical lighting across rooms. In addition, the researcher wears a mobile lapel microphone to aid in distinguishing speakers (ie, researcher speech from participant speech).

##### Researcher Observational Ratings

The ADOS-2 [41] is a widely used gold-standard diagnostic instrument for autism in which a trained administrator leads a series of activities designed to elicit social interaction. The administrator assigns scores on numerous ordinal scales within domains, such as language and communication, reciprocal social interaction, and stereotyped behaviors and restricted interests. In this study, as per the LEAP protocol, we used modules 3 and 4 activity schedules, which were designed for individuals who are verbally fluent.

##### State Anxiety

The 6-item short form of the Spielberger State-Trait Anxiety Inventory state scale [78] comprises the 6 items exhibiting the highest item-remainder correlations in the full scale. The short form displays excellent correlation with full-scale scores (*r*≥0.90), and acceptable reliability and validity. Participants rate items (eg, I feel calm) on a 4-point scale between 1 (not at all) and 4 (very much) according to how they feel “right now.”

### Remote Measurement

#### The RM System

##### Overview

The RM system comprises 4 components encompassing both active and passive data collection for a period of 28 days. This period was chosen pragmatically, considering the trade-off between gathering sufficient data to enable target analyses and acceptability to participants. Active data collection is via the RADAR aRMT app and MyJournE (Digital Marmalade) mood monitoring app. Passive data collection is via the RADAR pRMT app, Fitbit Inspire 2 wristband, and Fitbit app. Both active and passive data acquisition components are described in detail in subsequent sections. Figure 1 shows how multimodal data from the RM system map onto the domains of interest for measure development.

##### Active Components

We will use the RADAR aRMT primarily to administer our questionnaire schedule with prompts sent by push notification. The schedule includes a daily sleep quality item, biweekly attention items, and fortnightly social communication items (instruments detailed in subsequent sections). We will also use the aRMT to provide information about weekly progress, informing participants when each of the 4 weeks has been completed. Finally, at the end of the 28 days, system usability and user experience items will be administered to quantitatively capture participant satisfaction with the system. The schedule for the active components is summarized in Table S2 in Multimedia Appendix 1, and the instruments used are detailed in subsequent sections.

MyJournE is a mood monitoring app codesigned by young people [79] and researchers at KCL and developed by a UK-based software development company Digital Marmalade [80]. The app sends reminders via push notification twice per day for participants to complete the mood logs. These include a series of questions to efficiently capture context (eg, who a participant is with, where they are, and what they are doing), followed by a general happiness rating on a 5-point scale from very unhappy to very happy. This is followed by an implementation of the Positive and Negative Affect Scale (PANAS; refer to subsequent sections) in which each item is accompanied by a visualization of the emotion word involving a purple bean character (Figure S3 in Multimedia Appendix 1). Participants respond using a movable visual slider. Visual supports are commonly used by autistic people to aid in processing abstract concepts [81] and thus may contribute to acceptability, especially in the context of high rates of alexithymia among autistic individuals.

##### Passive Components

Fitbit Inspire 2 is a commercial fitness tracking device that records heart rate via photoplethysmography and motion via an accelerometer. It is lightweight, unobtrusive, and allows for a choice of strap material; thus, it is well-placed to cater for some degree of sensory sensitivity. Using proprietary algorithms, Fitbit provides heart rate, step count, and sleep staging data. Fitbit models manufactured after 2017 estimate time spent in 4 sleep stages (awake, light sleep, deep sleep, and rapid eye movement) using machine learning methods applied to motion and heart rate [82]. From this, sleep parameters such as total sleep time and wake after sleep onset may be derived [49].

The RADAR pRMT collects a variety of Android smartphone sensor data without the need for regular participant input. Specifically, these data include physical motion (eg, from the onboard accelerometer and gyroscope), ambient light levels, GPS location, number of Bluetooth-enabled devices in range, phone and app use data, and battery levels. GPS coordinates are offset by a random number for each participant to obscure the absolute location while preserving the relative location (ie, information about distance, duration, and direction of travel). In addition, for this project, we used a custom build of the app [83] that included functionality to access call and SMS logs including data on incoming and outgoing call duration, SMS length, and whether they were with a saved contact. These data are potentially relevant in capturing aspects of social functioning and, due to Google Play Store restrictions, are not included as standard in the app. No call or SMS content is recorded, and participants are able to freely grant or remove permissions for different data sources in the app settings.

#### Procedure

Given the novelty, complexity, and privacy considerations of RM data collection, our community representatives stressed the importance of a comprehensive information process with a clear description of which information is collected, how it is managed, and how it will be used. With this in mind, we developed an extended information and consent process. Eligible participants will be sent an email with attachments comprising the participant information sheet and a participant handbook (Multimedia Appendix 2). The participant handbook contains additional information on the types of data collected, particularly those that may feel more personal (eg, location and app use data), and how the data are pseudonymized, stored, and processed. Participants are also invited to a study information video call, which is typically scheduled at least 1 week after the email to allow time for reading the information documents. The call takes approximately 30 minutes, aims to run through the study information at a suitable pace, and allows time for questions. At the end of the call, participants who are happy to proceed are provided with a link to a web-based consent form, which they may complete as part of the call or in their own time. Consenting participants are invited to an enrollment video call approximately 1 week after the information call to allow for a Fitbit to be posted to their home.

The enrollment call takes approximately 1 hour and comprises app installation, setup, and Fitbit connection. The MyJournE app, RADAR active app, and the Fitbit app are installed from the Google Play Store whereas the custom RADAR passive app is installed from GitHub to the Android Application Package. Participants are given log-in details for each app and given a chance to explore the active data collection apps. The RADAR passive app requests access to several smartphone data sources. Participants are taken through these and reminded that they can control the data the app accesses by toggling permissions and are free to disallow access to any data source at any time if they feel uncomfortable. Finally, participants are taken through setting up their Fitbit device and pairing it with the app.

The 28-day data collection period starts the day after the enrollment call. Participants receive reminders to respond to the active components. A day 3 check-in is administered via video or phone call or email to check if the participant is happy with how the month is proceeding and to troubleshoot any issues. Researchers complete regular checks by accessing the RADAR-base platform to ensure data are coming in for each component (ie, Fitbit, pRMT app, aRMT app, and MyJournE). We contact the participant or relevant technical support teams for troubleshooting if any data component is not received or largely missing. For example, if data checks show that Fitbit data are missing, the participant is contacted to establish if the device is being worn or if there are syncing or charging issues. If the participants report that they are finding the device uncomfortable, an alternative strap is offered if it is felt that this may help. If data are missing from the smartphone apps, similar troubleshooting occurs, for example, to establish if notifications are being received or if the participant is simply finding it difficult to respond.

At the end of the 28 days, participants are notified to complete the user experience questionnaires via the RADAR aRMT. These questionnaires are available for 1 week, after which data are no longer collected via the aRMT app. Participants are also invited to complete a feedback interview during the week following completion. The feedback interview is conducted via a video call and lasts around 1 hour. It also includes questions eliciting participant opinions on the study information and enrollment processes, general experience and impact on daily life (including discussion of potential Hawthorne effects), Fitbit usability and comfort, active app scheduling, and passive data collection acceptability. Participants keep the Fitbit device at the end of the month and receive £20 (US $26.81) in Amazon vouchers for their time spent taking part in the feedback interview.

#### Measures

##### Autism Ratings

The Social Responsiveness Scale Short Form [84] is a questionnaire measure of autistic characteristics, particularly, in the social communication domain. It comprises 16 items derived from the full parent report version (65 items). The short form showed excellent reliability (α=.96) and substantial associations with social features of autism measured by gold-standard instruments (eg, Autism Diagnostic Interview-Revised Social subscale, *r*=0.48, and ADOS-2 module 4 Social Affect, *r*=0.49) [84]. In this study, it is administered as a self-report questionnaire on days 8 and 15 of the 28-day RM period.

##### Inattention and Hyperactivity Ratings

On the basis of a previous experience sampling study [85], we used a 4-item scale comprising 3 inattention items (eg, “I was easily distracted and could not concentrate over a longer period of time”) and 1 hyperactivity item (“I felt restless or fidgety”), based on *DSM-5* ADHD criteria. In a community sample of adolescents aged between 14 and 17 years, internal consistency for the inattention items was satisfactory (α=.67-.79) [85]. In this study, items are rated every 4 days on a scale from 0 (not at all) to 100 (all the time) using a movable slider.

##### Sleep Quality Ratings

The Single-item Sleep Quality Scale [86] is a simple measure of overall sleep quality rated on a ten-point scale. It shows a strong association with widely used questionnaire measures and was able to differentiate normal, borderline, and problem sleepers in a study of patients with depression [86]. In this study, participants are asked to rate their sleep quality for the night before each morning on a scale from 0 (terrible) to 100 (Excellent) using a moveable slider. The 0-100 scale was used for consistency with the inattention and hyperactivity items.

##### Mood Ratings

The PANAS is a widely used 20-item measure of positive and negative affect in which participants rate the intensity with which they are feeling each of 10 positive and 10 negative mood descriptors (eg, “enthusiastic” and “distressed”). Internal consistency for both scales was shown to be excellent (α=.84-.90) [87]. In the MyJournE app, items are rated according to the question “How appropriate is this word today?” using a 5-point movable slider scale ranging from “very slightly/not at all” to “extremely.”

##### User Experience Ratings

The System Usability Scale [88] is a 10-item usability questionnaire in which statements (eg, “I thought the system was easy to use”) are rated on a 5-point Likert scale from 1 (“strongly disagree”) to 5 (“strongly agree”). The scale has been demonstrated to have excellent internal consistency (α=.91) [89]. In this study, participants are notified via the RADAR aRMT app to complete the System Usability Scale based on their experience of the RM system *overall* (Fitbit and smartphone apps) on day 29 after enrollment. At the same time, participants were also notified to complete a separate user experience questionnaire with 7 items specific to the Fitbit and 10 items specific to the active questionnaire apps [90].

### Ethical Considerations

Ethics approval was obtained for LEAP and LEAP epilepsy groups at KCL and UCAM from the London-Central and Queen Square Health Research Authority Research Ethics Committee (13/LO/1156) and at RUMC from the RUMC’s Medisch-Ethische Toetsingscommissie (2019-5942; NL72033.091.19). For the RM comparison group, KCL Research Ethics was obtained (LRS/DP-23/24-42359). Compliance with European and UK data protection policies was ensured by performing a data protection impact assessment in consultation with the KCL information compliance department and compliance teams at local sites. RM data are pseudonymized via a study identification code, and dummy Fitbit accounts are set up for participants, which are not linked to their personal information. Data acquired from the Fitbit and smartphone apps are automatically encrypted and uploaded to secure servers as part of the RADAR platform. Video and other identifiable data are kept separately on secure servers and shared across study sites for analysis purposes with explicit consent.

### Planned Analyses

#### Overview

This protocol is primarily intended to support evaluation of the acceptability and feasibility of the range of candidate technologies and methods that it uses. Secondarily, it may provide rich, multimodal data supporting the exploration of a range of potentially meaningful outcome measures. The potential avenues for such exploration are numerous and dependent not only on the quality of the resulting data but also on available data processing tools and analytic approaches, which are expected to evolve rapidly. Therefore, in this paper we describe our primary analyses of acceptability and feasibility in detail and give only several examples of candidate outcome measure exploration planned in this study. The specifics of these secondary analyses are beyond the scope of this paper but will be presented in individual outputs. Our featured initial domains for outcome measure development are based on community priorities (eg, sleep and mental health) [91,92], tractability based on currently available analytic tools, and innovation in terms of building on existing work. Initial data processing plans are provided in Table S3 of Multimedia Appendix 1.

#### Acceptability and Feasibility

##### Overview

Acceptability and feasibility will be evaluated comprehensively via quantitative and qualitative analyses (Table 2). Uptake and completion rates will be calculated at the level of each system (ie, augmented ADOS-2 and RM protocols), whereas quantification of adherence, tolerance, and participant experience will differ according to subcomponents; for example, adherence to wearing the Fitbit is examined separately to adherence to questionnaire completion. Qualitative data, for example, reasons for declining to participate and for withdrawal will contribute to our understanding of quantitative information and, in the case of our RM protocol, will support a larger qualitative framework analysis of feedback interviews. Similarly, feasibility will be evaluated with reference to participant eligibility, data availability, and data quality and may be specific to each analytic approach. Limiting factors for the proportion of usable data will be identified; for example, establishing whether active RM data are missing due to technical failure (eg, notifications not sending) or nonresponse.

**Table 2:** Planned metrics for acceptability and feasibility.

		In-person components (E4 and video recording)	Remote measurement components (Fitbit, pRMT^a^, aRMT^b^, and MJE^c^)
**Acceptability**			
	Uptake	Proportion consenting and reasons for decline	Proportion consenting and reasons for decline
	Adherence and tolerance	E4 wear time and reasons for removal and requests for discontinuation of filming	Fitbit wear time and reasons for removal, pRMT app permissions granted, and aRMT and MJE response rates
	Experience	STAI State-6^d^ (before and after assessment)	Usability ratings (SUS^e^ and UEQ^f^) and feedback interview (qualitative)
	Completion	Proportion completing protocol and reasons for withdrawal	Proportion completing protocol and reasons for withdrawal
**Feasibility**			
	Eligibility	Proportion eligible and reasons for ineligibility	Proportion eligible and reasons for ineligibility
	Data availability	Missing data and reasons for missingness (eg, equipment malfunction)	Missing data; reasons for missingness (eg, hardware and software malfunction versus acceptability issue)
	Data quality	Proportion of usable data for each analysis and reasons for low-quality data	Proportion of usable data for each analysis and reasons for low-quality data

^a^pRMT: passive remote measurement technology.

^b^aRMT active remote measurement technology.

^c^MJE: MyJournE app.

^d^STAI State-6: 6-item short form of the Spielberger State-Trait Anxiety Inventory.

^e^SUS: System Usability Scale.

^f^UEQ: User Experience Questionnaire.

##### Predictors of Acceptability and Feasibility

Predictors of certain acceptability and feasibility metrics will be explored. Primarily, differences between autistic and nonautistic groups will be examined for each metric with formal parametric and nonparametric statistical methods applied where possible. Age, cognitive ability, and adaptive ability will be assessed as predictors of uptake, adherence (wear time and active app response rates), usability ratings, and completion. We will also explore the association between Fitbit wear time and the tactile sensitivity subscale of the Short Sensory Profile [93] completed by participants as part of the main LEAP study.

#### Planned Exploration of Candidate Outcome Measures

##### Digitally Augmented ADOS-2 Data

Audiovisual data from ADOS-2 will be processed using machine learning–based tools, assisted by researcher annotation of key behaviors. We will explore associations between resulting features extracted from the audiovisual data and observer-assigned scores in key domains. In so doing, we will explore the performance of existing speech and video processing tools and the suitability of extracted features for quantifying clinically relevant characteristics via more objective, precise, automatically generated scores. Note that it is not our intention to use these data to explore diagnostic classification approaches as has been done elsewhere. Our focus is on developing tools to quantify characteristics that may be used to understand heterogeneity within clinical classification and to evaluate interventions. For example, drawing on previously published pipelines [58], our speech data will be processed using open-source, automated diarization, transcription, and conversation analysis tools such as pyannoteAI Audio [59], OpenAI Whisper [60], and ConvoKit [61], respectively (Table S3 in Multimedia Appendix 1). Resulting features (eg, number of conversational turns and the length of participant and researcher utterances) will be tested for association with ADOS-2 language and communication codes indicating the amount and nature of social speech and reciprocal social interaction codes for the frequency and quality of social initiation and response. Such associations will be tested using conventional regression-based statistical analyses.

In addition, we will explore the ability of machine learning and computer vision tools to identify instances of verbal and nonverbal behaviors relevant to ADOS-2 scoring algorithms. For example, drawing on existing work identifying and classifying stereotyped movements from ADOS-2 data in children [62], we will explore the feasibility of applying such methods to our adolescent and adult data, as well as assessing the utility of integrating E4 motion and psychophysiological data to refine classification.

Using Empatica E4 photoplethysmography and EDA data, we plan to develop pipelines for investigating autonomic arousal in the context of the ADOS-2 assessment. Data will undergo quality control procedures (eg, [63,94]) and processing (Table S3 in Multimedia Appendix 1). Tasks will be demarcated using event markers generated via hand waves. Key tasks of interest within the ADOS-2 will be identified, that is, those hypothesized to have high (presenting a story from a cartoon and discussing emotions) versus low (completing a puzzle and describing a picture) psychosocial demand for autistic people. Within each relevant task, heart rate, heart rate variability, and EDA features will be calculated, applying and testing existing pipelines [95,96]. Derived indices of autonomic arousal will then be compared across conditions within participants. If data are of sufficient quality and power, linear mixed effects models (LMEMs) will investigate group differences in autonomic response to psychosocial demand [47]. Association of autonomic responses with subjective state anxiety scores from the beginning and end of the assessment will also be explored.

##### RM Data

###### Overview

Using RM data, we will focus on exploring objective, multimodal indices from passive data in several key domains (sleep, mental health, and social functioning) and investigate their associations with corresponding self-report data. Due to the relatively short observation period and lack of planned intervention during that time, this exploration will focus on between-subject analyses as opposed to within-subject change over time. Given the hierarchical data structures produced by repeated measurement, associations will be investigated using LMEMs. These account for dependent data points flexibly, with minimal loss of power [97].

###### Missing Data

Given the challenges associated with collecting data remotely, we anticipate missing data due to varying participant engagement levels, device issues, or network connectivity. We will draw on published research establishing feature-specific guidance on acceptable levels of missing data to determine data eligibility for further analysis [98]. Where possible, we will use multiple imputation to account for missing values (eg, where certain modalities have some missing values for a participant in a multimodal analysis). Coupled with this, LMEMs allow for retention of cases with missing observations and are compatible with certain maximum likelihood–based estimation procedures [99]. Multiple imputation– and maximum likelihood–based approaches are considered unbiased where data are missing at random [100]. The plausibility of this assumption will be assessed on a case-by-case basis and be addressed where necessary, for example, using sensitivity analyses.

###### Sleep

As well as drawing on Fitbit-provided sleep staging data, we will incorporate passive smartphone data, such as light levels, phone motion, and app use, to derive objective indices of sleep architecture. This novel approach is intended to improve accuracy in differentiating periods of sleep versus wakefulness. In particular, we will target sleep parameters identified commonly as disrupted in autistic youth and adults, for example, sleep onset latency, wake after sleep onset, sleep efficiency, and percentage of rapid eye movement sleep [33,101]. We will test the associations between these objective parameters and daily subjective sleep quality ratings using LMEMs. In addition, we will use cluster analysis to identify more homogeneous, objectively recorded sleep patterns.

###### Mental Health

Drawing on existing research into smartphone sensor-based indicators of mental health, for example, from location and activity data, phone and app use patterns, and call and SMS logs [102], we plan to test currently available tools and procedures for feature extraction and assessment of correspondence between passive data and subjective positive and negative affect as reported in the PANAS, administered via the MyJournE app (eg, [103]). We will also explore associations between extracted features and mental health measures collected as part of the LEAP questionnaire battery (ie, the Beck Depression Inventory [104] and Beck Anxiety Inventory [105]) and reported diagnoses.

###### Social Functioning

Finally, we plan to use similar methods to explore aspects of everyday social functioning and environments that may be indexed in our passive RM data. Our data offer an opportunity to explore objective indices of social functioning and environment in everyday life in relation to subjective reports of social communication, social activities, and mood. We will adapt pipelines developed in other populations to index various aspects of social functioning, including social activity [106], “sociability” (comprising communication and social exploration domains) [51], and loneliness [52], from multimodal data (including duration, frequency, and diversity of telephone calls, Bluetooth connectivity, GPS location, and smartphone screen interactions). We aim to develop similar multimodal indices that may be referenced against subjective measures, such as the Vineland Adaptive Behavior Scales, Social Responsiveness Scale Short Form, and the MyJournE context questions (eg, “Who are you with?”).

## Results

Following ethics approvals documented earlier, the first participant was enrolled for the digitally augmented ADOS-2 component on September 11, 2021, and for the RM component on March 3, 2022. Data collection within the LEAP and LEAP epilepsy samples is complete as of October 2024. In the digitally augmented ADOS-2 component, 190 participants took part (LEAP: n=167, 87.9% and LEAP epilepsy: n=23, 12.1%). In the RM component, 86 participants have been enrolled (LEAP: n=47, 55%, LEAP epilepsy: n=14, 16%, and RM comparison: n=25, 29%). In the RM comparison group, data will be collected in an ongoing manner until August 2025.

Several planned analyses are underway. In our primary outcome domains of acceptability and feasibility, qualitative framework analysis coproduced with community members is in its final stages and will contribute to a key planned mixed methods publication on the acceptability and feasibility of RM. This publication will also outline work with autism community members during initial stages and the impact on the protocol. Quantitative acceptability and feasibility analyses contributing to this publication are also underway; for example, we have explored adherence to Fitbit wearing and the active questionnaire schedule. Elsewhere, we have developed working pipelines for analysis of ADOS-2 speech data and RM sleep data.

## Discussion

### Study Contributions

Autistic people face diverse and complex challenges in daily lives that are difficult to quantify using conventional techniques. Measuring treatment response in a scalable, precise, and unbiased manner is particularly problematic, and a lack of tools available to do this has impeded progress in evaluating autism-specific interventions for such challenges. Development of better measurement tools for quality of life, mental health, social well-being, and sleep was among the top 5 priorities identified by autistic adults and other stakeholders in the study by Benevides et al [91]. Digital technology offers an array of compelling opportunities to measure more objectively, with lower burden and greater ecological validity. This study protocol was designed with input from autism community representatives to develop and evaluate acceptable digital measures relevant to priority outcomes. Our dual in-person and remote approach enables us to develop an understanding of how such measures may relate to and potentially complement each other in the context of a range of study designs. If demonstrated to be adequately acceptable and feasible, the measures explored in this study could be used to enhance understanding of everyday challenges for autistic people. They could support clinical research by providing outcome measures that are specific and responsive to small changes in meaningful outcomes.

Most existing studies applying digital technology to challenges in the diagnosis and management of autism are aimed at proof-of-concept and have relatively small sample sizes [107]. Understanding generated from this study of digital acceptability and feasibility could facilitate the application of digital measures at scale and assist implementation of digital supports for autistic people, for example, app-based mental health interventions [108] and wearable devices that may help autistic people to understand and communicate their emotions [109] or predict maladaptive behaviors [77].

### Potential Strengths and Limitations

Data collection in the LEAP study is well positioned for achieving these aims. The sample is already well characterized, meaning that factors potentially associated with acceptability and feasibility may be thoroughly investigated. LEAP includes high-quality measures of autism clinical characteristics, intellectual ability, and co-occurring symptoms, factors that may influence participant uptake and experience of digital measures. Certain candidate measures may also be validated against robust conventional measures in domains of interest, for example, gold-standard autism assessments and well-validated questionnaire instruments. For other novel measures, we do not incorporate a reference standard. In the domain of sleep, polysomnography is not included because its feasibility for implementation outside of clinical settings is problematic and its representativeness in capturing everyday sleep is unclear. However, we do incorporate a daily subjective sleep quality measure, which is an important clinical end point.

For the RM component, instruments were selected to build a system that maximizes acceptability and accessibility (within the context of independent smartphone users). Accordingly, we selected very brief active measures to minimize participant daily time burden and interference with everyday life. We also selected Fitbit Inspire 2 as our wearable device of choice, given its widespread use in the broader population, unobtrusive design, and affordability. However, this approach comes with challenges in terms of data quality and analytic potential. Both wearable devices use photoplethysmography to estimate heart rate, rather than obtaining direct measures via electrocardiography. Use of Fitbit also necessitates relying on proprietary algorithms for heart rate, step count, and sleep pattern, which limits flexibility in analysis. On the other hand, Fitbit provides ready-to-use processed data with established performance against gold-standard measures [82,110,111]. Across studies, recent-generation Fitbit models have been shown to have high sensitivity (0.95-0.96) in identifying sleep epochs, lower specificity (0.58-0.69), and a tendency to underestimate sleep onset latency [82]. Despite efforts to maximize the acceptability of remote data collection, some level of noncompliance is expected, and understanding factors associated with these forms a primary area of investigation for this study.

Another limitation is that the focus on independent smartphone users is likely to exclude many participants with intellectual disabilities, a group forming a considerable proportion of autistic people [112]. Our hope is that the current research may identify promising candidate measures that could be adapted for populations with ID. Indeed, enhancing our understanding of how objective, passive measures relate to subjective experience may pave the way for the development of novel measures of internal states, such as anxiety, which do not require explicit self-report [113]. Similarly, our inclusion criterion of “willingness to try to wear the Fitbit Inspire 2 as much as possible for 28 days” may exclude participants with particular sensitivity to touch. However, our selection of the Fitbit Inspire 2 was largely based on discussion with autistic community members who advised us that selecting a lightweight device with a choice of strap material may minimize exclusion of those with sensory sensitivities and support adherence. Furthermore, our explicit inclusion of questions on device comfort in the feedback interview is intended to advance understanding of the factors that may influence adherence to wearing such a device for autistic participants in research studies.

More generally, our sample is primarily derived from LEAP, a volunteer sample that has been further selected based on eligibility criteria mentioned earlier. This limits the generalizability of the current findings to the wider, highly heterogeneous autistic population. However, it is hoped that responding to acceptability and feasibility challenges highlighted by this study will inform future research that may be more widely applicable. In turn, highly precise, objective, digital end points may ultimately contribute to identifying subgroups of the autistic population that may reflect different mechanisms underpinning challenges and different responses to intervention.

Data privacy is another important issue raised in our community involvement activities and those of others [114]. Digital technology is powerful in the sense that large quantities of data capturing potentially sensitive features are generated with low effort and awareness. Research has shown that most owners of smart devices are willing or would consider sharing their digital data for health research purposes [115]. However, for certain others, this constitutes an uncomfortable invasion of privacy. In the general population, acceptability may depend on how data are used (eg, for the good of the public), managed (eg, using anonymization procedures), and with whom they are shared [116]. This study has the potential to provide insight into the circumstances under which such data collection is acceptable for autistic people and how to communicate effectively about data use and sharing.

Finally, although this is an observational study, our RM procedures may cause people to change aspects of daily living, introducing Hawthorne effects. For example, use of the Fitbit for its intended purpose as a fitness tracker may lead to changes in activity levels and sleep habits, and knowledge that app use is recorded may cause a participant to change app use habits. Because a putative advantage of digital measurement (particularly, passive and objective measures) is a reduction in potential observation or attention effects in the context of clinical trials, it is important to investigate these during measure development. Therefore, we ask specifically about perceived behavioral changes in our qualitative interview. However, quantitative investigation of within-subject variance over time is out of scope in our study due to the relatively short observation period. Future studies may wish to draw on platforms providing the capability to blind participants from on-device information and app-based dashboards showing summarized data (eg, Oura Ring [117]).

### Conclusions

Despite the range of potential challenges in digital measure development, particularly in the field of autism research, identification and understanding of these are incorporated as key aims of this study. Our collection of both quantitative and qualitative data on acceptability will allow a more thorough understanding of factors influencing uptake, adherence, completion, and, importantly, experience of the protocol. Careful consideration of the acceptability and feasibility of candidate measurement techniques, alongside extracting and validating meaningful indices of outcomes important to community stakeholders, is essential in developing measures that live up to the potential afforded by digital innovation.

## Appendix

Multimedia Appendix 1 Additional tables and figures comprising Autism Diagnostic Observation Schedule-2 (ADOS-2) pandemic–related adaptations, schedule of events for the 28-day Mobile Measures Month, Empatica E4 device set-up, videocamera and lapel microphone set-up for the ADOS-2, screenshots from the MyJournE app, and additional detail on proposed data processing for passive data collection modalities.

Multimedia Appendix 2 Participant handbook for the remote measurement component.

## Abbreviations

ADHD: attention-deficit/hyperactivity disorder
ADOS-2: Autism Diagnostic Observation Schedule-2
aRMT: active remote measurement technology
DSM-IV: Diagnostic and Statistical Manual of Mental Disorders (Fourth Edition)
DSM-5: Diagnostic and Statistical Manual of Mental Disorders (Fifth Edition)
EDA: electrodermal activity
ICD-10: International Statistical Classification of Diseases, Tenth Revision
JAKE: Janssen Autism Knowledge Engine
KCL: King’s College London
LEAP: AIMS Longitudinal European Autism Project
LMEM: linear mixed effects model
PANAS: Positive and Negative Affect Scale
pRMT: passive remote measurement technology
RM: remote measurement
RUMC: Radboud University Medical Centre, Nijmegen
UCAM: University of Cambridge
