# Supplementary material for: A Dual In-Person and Remote Assessment Approach to Developing Digital End Points Relevant to Autism and Co-Occurring Conditions: Protocol for a Multisite Observational Study
Source: JMIR Res Protoc. 2025 Oct 3;14:e71145. doi: 10.2196/71145 (PMC12534762; doi:10.2196/71145)
Supplement: Multimedia Appendix 1 [file resprot_v14i1e71145_app1.docx]

Multimedia Appendix 1

Table S1. Pandemic-related adaptations for ADOS-2 informed assessment procedures, based on suggestions by Emma Woodhouse (personal correspondence).

| Task | Adaptation |
| --- | --- |
| Throughout | Social distancing should be used as advised by government/local requirements. Clear masks and/or Perspex screen may also be used to minimise spread of disease, according to government/local requirements and/or participant/researcher preference. |
| Construction task | Due to distancing you will not be able to put the blocks within reach and create a barrier with your arm. Keep back several blocks back and stick to the same instructions (“let me know if you need some more”) and observe how they indicate that they need more blocks. |
| Make believe and joint interactive play | Several items have been removed due to contamination concerns. Ensure the child has two of the figures and the holographic disc, along with some of the other items. Keep back one action figure and a few items for yourself. When you join in with the play, incorporate the distance between you within your ideas (e.g. “I’m stuck on a different planet and I need to get back to your planet, how are we going to do this?) |
| Demonstration task | Administer as described in the manual. If they are unable to complete the toothbrushing task and the handwashing task, do not move onto the ‘real’ objects (towel and soap) – these have been removed due to contamination concerns. |
| Description of a picture | Use the instructions as described in the manual. The only difference is that you won’t be able to look at this jointly. |
| Telling a story from a book | Use the instructions as described in the manual. Explain that you will both have the same book and will tell the story together. Ensure that you start by describing the first page, and make sure they are following when you turn over to the next page. Do not be overly directive about when they should turn their page. Observe how they co-ordinate this joint task (e.g. when they are telling the story, are they ‘checking in with you’ to ensure you are following / are on the same page). As described in the manual, ensure you take a turn before the end of the story. |
| Cartoons | No change from the manual. Try to prevent the child from touching the cards. |
| Conversation & Reporting | No change from the manual. |
| Emotions | No change from the manual. |
| Social difficulties and annoyance | No change from the manual. |
| Break | Some items have been removed due to contamination concerns. Other than this, administer as described in the manual. |
| Friends, relationships and marriage | No change from the manual. |
| Loneliness | No change from the manual. |
| Creating a story | Keep back the following five items for the child: miniature playing card, paperclip, lollystick, cocktail umbrella, cardboard disc. From the remaining items, select five items as described in the manual (two objects that are used in the way they were intended, three objects that will be used in a novel or creative, one of the five objects must be used as the agent or actor in the story. Make up a story using your five items, then ask them to make up a story with their five items. Ensure that you do not give the items to the child until after you have finished your demonstration story. Dispose of the miniature playing card, paperclip, lollystick, cocktail umbrella, cardboard disc immediately after the assessment. |

Table S2. Schedule of events and active reporting for the 28-day Mobile Measures Month

|  |  | Days post-enrolment | | | | | | | | | | | | | | | | | | | | | | | | | | | | | |
| --- | --- | --- | --- | --- | --- | --- | --- | --- | --- | --- | --- | --- | --- | --- | --- | --- | --- | --- | --- | --- | --- | --- | --- | --- | --- | --- | --- | --- | --- | --- | --- |
|  | Pre | 0 |  |  |  |  |  |  | 7 |  |  |  |  |  |  | 14 |  |  |  |  |  |  | 21 |  |  |  |  |  |  |  | 29+ |
| Screening call | X |  |  |  |  |  |  |  |  |  |  |  |  |  |  |  |  |  |  |  |  |  |  |  |  |  |  |  |  |  |  |
| Information call | X |  |  |  |  |  |  |  |  |  |  |  |  |  |  |  |  |  |  |  |  |  |  |  |  |  |  |  |  |  |  |
| Consent | X |  |  |  |  |  |  |  |  |  |  |  |  |  |  |  |  |  |  |  |  |  |  |  |  |  |  |  |  |  |  |
| Enrolment call |  | X |  |  |  |  |  |  |  |  |  |  |  |  |  |  |  |  |  |  |  |  |  |  |  |  |  |  |  |  |  |
| Technology usage |  | X |  |  |  |  |  |  |  |  |  |  |  |  |  |  |  |  |  |  |  |  |  |  |  |  |  |  |  |  |  |
| Routine activities |  | X |  |  |  |  |  |  |  |  |  |  |  |  |  |  |  |  |  |  |  |  |  |  |  |  |  |  |  |  |  |
| Non-routine activities |  | X |  |  |  |  |  |  |  |  |  |  |  |  |  |  |  |  |  |  |  |  |  |  |  |  |  |  |  |  |  |
| Sleep routines |  | X |  |  |  |  |  |  |  |  |  |  |  |  |  |  |  |  |  |  |  |  |  |  |  |  |  |  |  |  | X |
| Daily mood logs |  |  | 2 | 2 | 2 | 2 | 2 | 2 | 2 | 2 | 2 | 2 | 2 | 2 | 2 | 2 | 2 | 2 | 2 | 2 | 2 | 2 | 2 | 2 | 2 | 2 | 2 | 2 | 2 | 2 |  |
| Daily sleep quality item |  |  | 1 | 1 | 1 | 1 | 1 | 1 | 1 | 1 | 1 | 1 | 1 | 1 | 1 | 1 | 1 | 1 | 1 | 1 | 1 | 1 | 1 | 1 | 1 | 1 | 1 | 1 | 1 | 1 |  |
| ADHD items |  |  |  |  |  | 1 |  |  |  | 1 |  |  |  | 1 |  |  |  | 1 |  |  |  | 1 |  |  |  | 1 |  |  |  | 1 |  |
| SRS Shortform |  |  |  |  |  |  |  |  |  | 1 |  |  |  |  |  |  |  |  |  |  |  |  | 1 |  |  |  |  |  |  |  |  |
| SUS |  |  |  |  |  |  |  |  |  |  |  |  |  |  |  |  |  |  |  |  |  |  |  |  |  |  |  |  |  |  | X |
| UEQ |  |  |  |  |  |  |  |  |  |  |  |  |  |  |  |  |  |  |  |  |  |  |  |  |  |  |  |  |  |  | X |
| Experience interview |  |  |  |  |  |  |  |  |  |  |  |  |  |  |  |  |  |  |  |  |  |  |  |  |  |  |  |  |  |  | X |

Notes. SRS: Social Responsiveness Scale; SUS: System Usability Scale; UEQ: User Experience Questionnaire


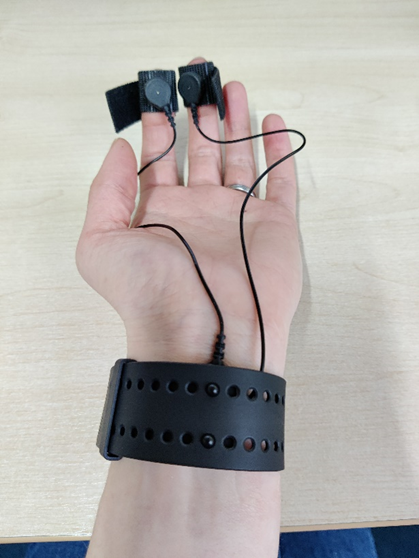

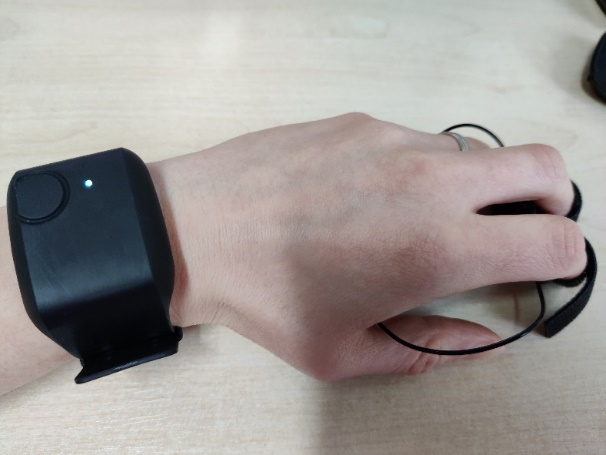


Figure S1. The Empatica E4 device with lead wire attachments


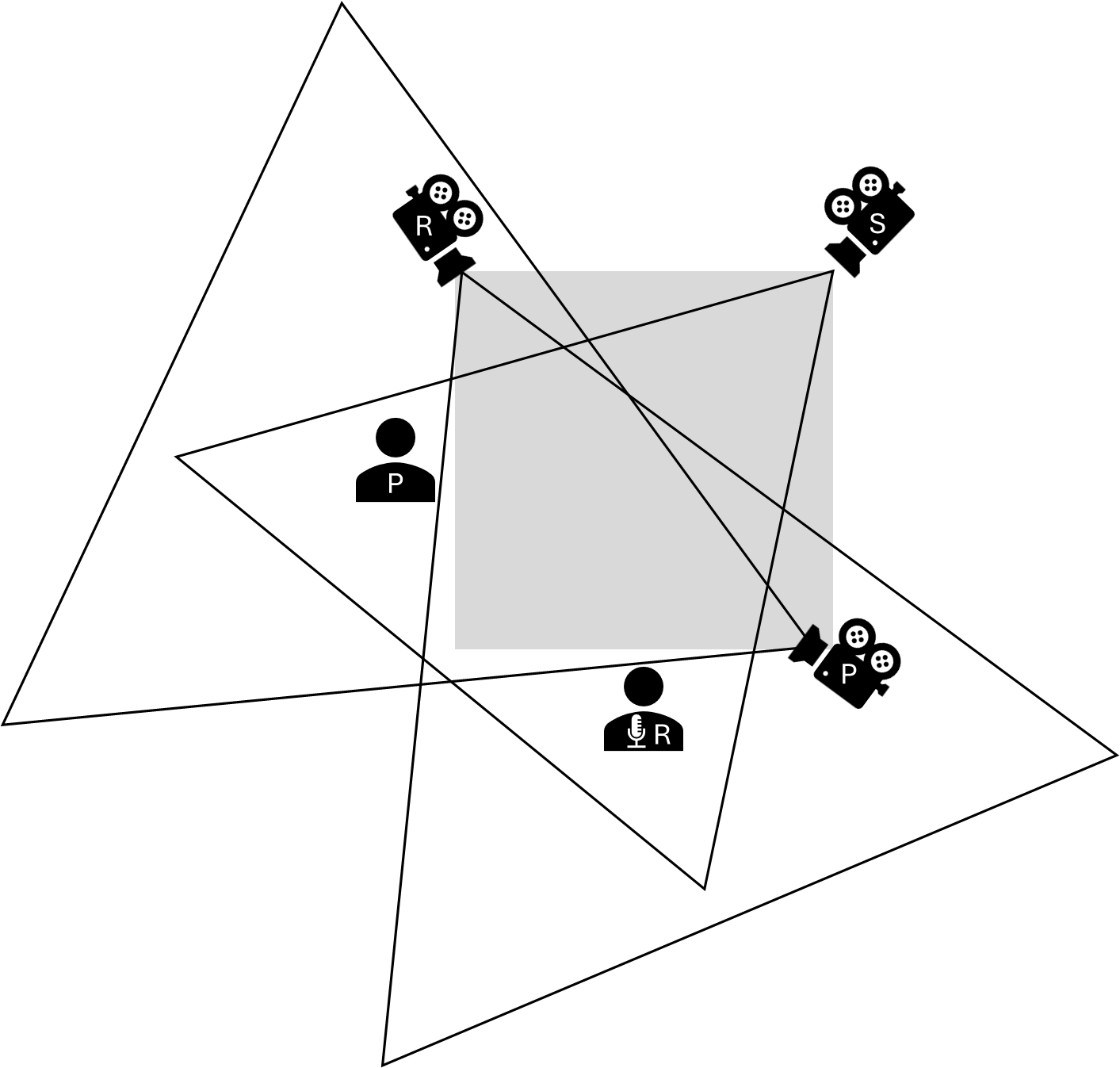


Figure S2. Schematic of the set-up for video and audio recording for the augmented ADOS-2. Notes. P denotes participant and participant-facing camera; R denotes researcher (the researcher also wears a lapel microphone) and researcher-facing camera; S denotes the camera capturing the overall scene.


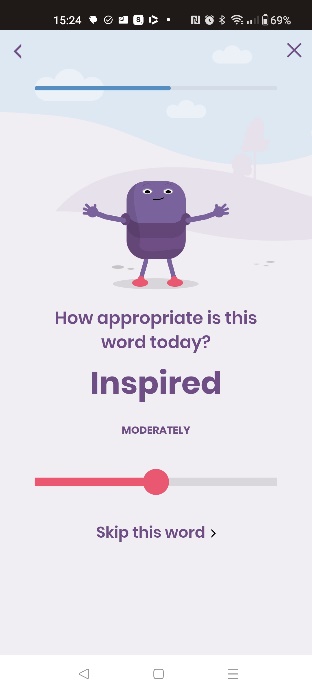

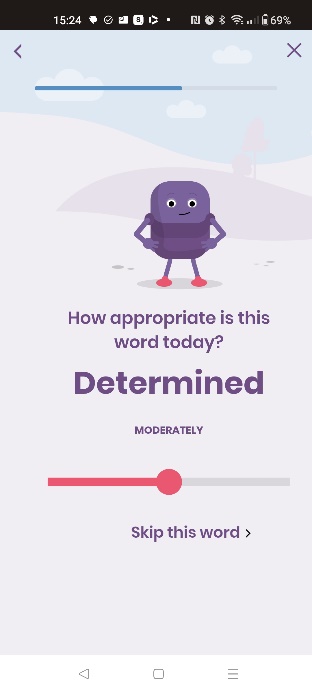

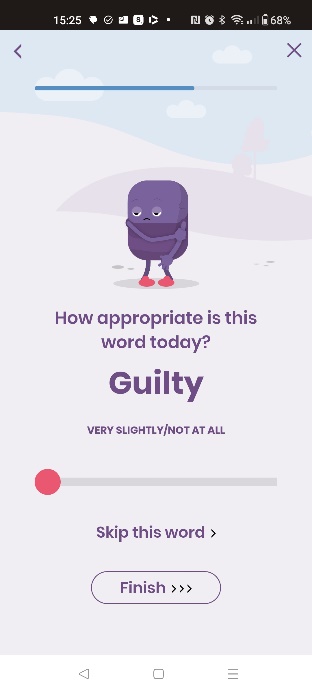

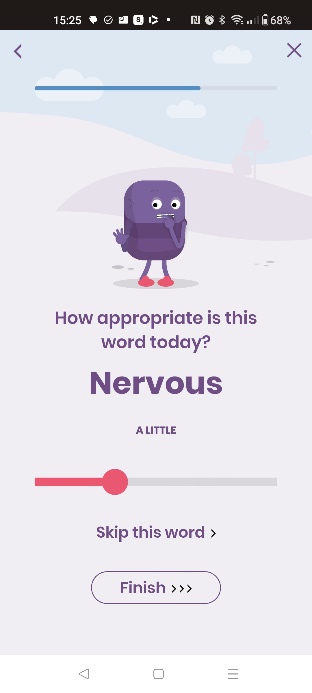

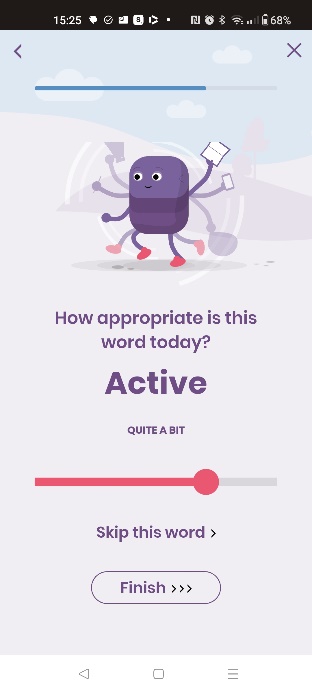


Figure S3. Screenshots from MyJournE

Table S3. Additional detail on proposed data processing for passive data collection modalities

| Raw data modality | Example processing tasks | Example tools | Example output | Potential challenges | Relevant citations |
| --- | --- | --- | --- | --- | --- |
| Audiovisual | Researcher annotation: -Manual diarisation to aid in training automatic diarisation tool (PyAnnote).  -Behaviours coded under D1 (sensory interest), D2 (mannerisms) and D4 (repetitive behaviours). | [VGG Image Annotator (VIA)](https://www.robots.ox.ac.uk/~vgg/software/via/) [1] | Timestamped annotation files (.csv; JSON) | -Manual annotation is time consuming and requires researcher training.  -Heterogeneity of behaviours coded (and not coded) under D1, D2, and D4 | [2,3] |
| Audio | -Automatic diarization including retraining of PyAnnote using our annotated data  -Automatic transcription  -Conversation analysis | -[PyAnnote](https://github.com/pyannote/pyannote-audio) [4] (diarization)  -[Whisper](https://github.com/openai/whisper) [5] (automatic speech recognition and transcription model),  -[ConvoKit](https://github.com/CornellNLP/ConvoKit) [6] (deep learning model for conversation analysis) | Quantitative conversation characteristics:  -number of turns  -length of utterance  -ratio of researcher to participant length of utterance | -Tools trained on neurotypical speakers  -Tools are trained in different environments (e.g. Pyannote is trained with telephonic conversations in which speakers are separated and have individual microphones).  -Existing models may not transfer well to the current study and tools produce high diarisation error rates. | [2] |
| Video | Pose estimation and automatic identification of stereotyped movements | [OpenPose](https://github.com/CMU-Perceptual-Computing-Lab/openpose) [7]  [PoseC3D](https://github.com/kennymckormick/pyskl/blob/main/configs/posec3d/README.md) [8]  [ASDPose](https://github.com/Dinstein-Lab/ASDMotion) [3] | -Movement in 2D space over time based on skeletal joint tracking  -Stereotyped movement confidence score | -Pipelines developed in different population and context (i.e., children completing lower ADOS-2 modules) may not transfer well to adolescents and adults completing predominantly module 4. | [3] |
|  | -Facial landmark tracking  -Head pose estimation  -Gaze estimation  -Facial Action Unit (FAU) recognition | [OpenFace](https://github.com/TadasBaltrusaitis/OpenFace) [9] | -Face detection confidence  -FAU presence and intensity scores  -Gaze angle values (relative to camera)  -Head pose co-ordinates | -Precision of gaze estimation expected to be low.  -FAU is sensitive to head pose variability and movement from speech  -Tools trained on neurotypical facial expressions may not capture autistic expressions adequately | [10–12] |
| E4: PPG Interbeat-interval data (IBI) | -Quality assessment  -Artifact removal (e.g., removing IBIs that differ from surrounding IBIs by more than 20% or those not physiologically plausible)  -Segmentation and labelling  -Handling missing data (e.g., apply missing beats threshold for discarding segment versus interpolation)  -Resampling will be considered, e.g., when combining EDA and PPG data. | Matlab ([Kubios HRV](https://www.kubios.com/)) [13]  [FLIRT](https://github.com/im-ethz/flirt) [14] | -Data quality indices  Heart rate (HR)  -min/max/mean HR  Heart rate variability (HRV)  -Root mean square of successive RR interval differences (RMSSD) | -Motion & loss of contact artifacts expected  -Due to the size of the dataset, it is not feasible to assess quality manually. Therefore, application of automatic artifact detection tools will be explored and applied. | [15,16] |
| E4: EDA | -Quality assessment (e.g., automatic artifact detection)  -Filtering (e.g., low-pass)  -Decomposition into tonic and phasic components  -Segmentation and labelling  -Resampling will be considered, e.g., when combining EDA and PPG data. | [EDAQA](https://github.com/iankleckner/EDAQA) [17]  [FLIRT](https://github.com/im-ethz/flirt) [14]  MatLab ([Ledalab](http://www.ledalab.de/)) [18,19]  [Awesome GSR](https://github.com/mintisan/awesome-gsr) | -Data quality indices  -Skin conductance level (SCL)  -Skin conductance response rate (SCRR) | -Motion & loss of contact artifacts expected  -Due to the size of the dataset, it is not feasible to assess quality manually. Therefore, application of automatic artifact detection tools will be explored and applied.  -Previous studies using E4 have largely used wrist as opposed to finger placement. Therefore, care will be taken to tailor data processing procedures when drawing on previous E4-specific algorithms. | [15,16] |
| Fitbit sleep data | -Identifying primary sleep periods in each 24-hour window  -Deriving sleep features for each primary sleep | Python packages:  -[pandas](https://pandas.pydata.org/) (dataset manipulation) [20]  -[miceforest](https://pypi.org/project/miceforest/) (multiple imputation) | Sleep features:  -Total sleep time  -Sleep onset latency  -Wake after sleep onset  -Sleep efficiency | -Working with data pre-processed by Fitbit proprietary algorithm.  -Data are subject to issues of unknown origin, such as overlapping sleep stages (i.e., periods of time simultaneously assigned to more than one sleep/wake stage).  -Missingness is anticipated, e.g., due to not wearing the device. | [21] |
| RADAR pRMT data | Deriving specific events relevant to sleep onset and offset from pRMT data sources:  -Accelerometer  -Light levels  -App usage  -Screen interaction | Python packages:  -[pandas](https://pandas.pydata.org/) (dataset manipulation) [20]  -[miceforest](https://pypi.org/project/miceforest/) (multiple imputation)  In-house testing:  -Change value thresholds associated with specific occurrences (e.g., phone pick-up, lights off) across multiple smartphone models. | Instances of  -lights off  -phone pick-up  -phone interaction | -Differentiating “lights off” from luminosity change for other reasons  -Differentiating phone pick-up from vibration and other movement  -Data are complex and variable across participants using different smartphone models  -Various sources of data missingness are expected  -Differing smartphone usage habits across participants makes data interpretation challenging | [22] |

**References for Table S3**

1. Dutta A, Zisserman A. The VIA annotation software for images, audio and video. MM 2019 - Proc 27th ACM Int Conf Multimed. Association for Computing Machinery, Inc; 2019;2276–2279.

2. O’Sullivan J, Bogaarts G, Schoenenberger P, Tillmann J, Slater D, Mesgarani N, et al. Automatic speaker diarization for natural conversation analysis in autism clinical trials. Sci Reports 2023 131. Nature Publishing Group; 2023;13(1):1–10. PMID: 37355730

3. Barami T, Manelis-Baram L, Kaiser H, Ilan M, Slobodkin A, Hadashi O, et al. Automated Analysis of Stereotypical Movements in Videos of Children With Autism Spectrum Disorder. JAMA Netw open. American Medical Association; 2024;7(9):e2432851. PMID: 39264628

4. Bredin H, Yin R, Coria JM, Gelly G, Korshunov P, Lavechin M, et al. pyannote.audio: neural building blocks for speaker diarization. ICASSP, IEEE Int Conf Acoust Speech Signal Process - Proc. Institute of Electrical and Electronics Engineers Inc.; 2019;2020-May:7124–7128.

5. Radford A, Kim JW, Xu T, Brockman G, McLeavey C, Sutskever I. Robust Speech Recognition via Large-Scale Weak Supervision. Proc Mach Learn Res. ML Research Press; 2022;202:28492–28518.

6. Chang JP, Chiam C, Fu L, Wang AZ, Zhang J, Danescu-Niculescu-Mizil C. ConvoKit: A Toolkit for the Analysis of Conversations. SIGDIAL 2020 - 21st Annu Meet Spec Interes Gr Discourse Dialogue, Proc Conf. Association for Computational Linguistics (ACL); 2020;57–60.

7. Cao Z, Hidalgo G, Simon T, Wei SE, Sheikh Y. OpenPose: Realtime Multi-Person 2D Pose Estimation Using Part Affinity Fields. IEEE Trans Pattern Anal Mach Intell. IEEE Computer Society; 2021;43(1):172–186. PMID: 31331883

8. Duan H, Zhao Y, Chen K, Lin D, Dai B. Revisiting Skeleton-based Action Recognition. Proc IEEE Comput Soc Conf Comput Vis Pattern Recognit. IEEE Computer Society; 2021;2022-June:2959–2968.

9. Baltrusaitis T, Zadeh A, Lim YC, Morency LP. OpenFace 2.0: Facial behavior analysis toolkit. Proc - 13th IEEE Int Conf Autom Face Gesture Recognition, FG 2018. Institute of Electrical and Electronics Engineers Inc.; 2018;59–66.

10. Parish-Morris J, Sariyanidi E, Zampella C, Bartley GK, Ferguson E, Pallathra AA, et al. Oral-Motor and Lexical Diversity During Naturalistic Conversations in Adults with Autism Spectrum Disorder. 2018;147–157. PMID: 33073267

11. Zampella CJ, Bennetto L, Herrington JD. Computer Vision Analysis of Reduced Interpersonal Affect Coordination in Youth With Autism Spectrum Disorder. Autism Res. John Wiley & Sons, Ltd; 2020;13(12):2133–2142. PMID: 32666690

12. Drimalla H, Scheffer T, Landwehr N, Baskow I, Roepke S, Behnia B, et al. Towards the automatic detection of social biomarkers in autism spectrum disorder: introducing the simulated interaction task (SIT). NPJ Digit Med. Nature Publishing Group; 2020;3(1):25. PMID: 32140568

13. Tarvainen MP, Niskanen JP, Lipponen JA, Ranta-aho PO, Karjalainen PA. Kubios HRV – Heart rate variability analysis software. Comput Methods Programs Biomed. Elsevier; 2014;113(1):210–220. PMID: 24054542

14. Föll S, Maritsch M, Spinola F, Mishra V, Barata F, Kowatsch T, et al. FLIRT: A feature generation toolkit for wearable data. Comput Methods Programs Biomed. Elsevier; 2021;212:106461. PMID: 34736174

15. Milstein N, Gordon I. Validating Measures of Electrodermal Activity and Heart Rate Variability Derived From the Empatica E4 Utilized in Research Settings That Involve Interactive Dyadic States. Front Behav Neurosci. Frontiers Media S.A.; 2020;14:566230. PMID: 33013337

16. Hu X, Sgherza TR, Nothrup JB, Fresco DM, Naragon-Gainey K, Bylsma LM. From lab to life: Evaluating the reliability and validity of psychophysiological data from wearable devices in laboratory and ambulatory settings. Behav Res Methods. Springer; 2024;56(7):1–20. PMID: 38528248

17. Kleckner IR, Jones RM, Wilder-Smith O, Wormwood JB, Akcakaya M, Quigley KS, et al. Simple, Transparent, and Flexible Automated Quality Assessment Procedures for Ambulatory Electrodermal Activity Data. IEEE Trans Biomed Eng. 445 HOES LANE, PISCATAWAY, NJ 08855-4141 USA: IEEE-INST ELECTRICAL ELECTRONICS ENGINEERS INC; 2018;65(7):1460–1467.

18. Benedek M, Kaernbach C. A continuous measure of phasic electrodermal activity. J Neurosci Methods. Elsevier; 2010;190(1):80–91. PMID: 20451556

19. Benedek M, Kaernbach C. Decomposition of skin conductance data by means of nonnegative deconvolution. Psychophysiology. John Wiley & Sons, Ltd; 2010;47(4):647–658. PMID: 20230512

20. pandas development team T. pandas-dev/pandas: Pandas [Internet]. Zenodo; 2024.

21. Zhang Y, Folarin AA, Sun S, Cummins N, Bendayan R, Ranjan Y, et al. Relationship between major depression symptom severity and sleep collected using a wristband wearable device: Multicenter longitudinal observational study. JMIR mHealth uHealth. JMIR Publications Inc.; 2021;9(4):e24604. PMID: 33843591

22. Rahimi-Eichi H, Coombs G, Bustamante CMV, Onnela JP, Baker JT, Buckner RL. Open-source longitudinal sleep analysis from accelerometer data (dpsleep): Algorithm development and validation. JMIR mHealth uHealth. JMIR Publications Inc.; 2021;9(10):e29849. PMID: 34612831
