# Supplementary material for: A Dual In-Person and Remote Assessment Approach to Developing Digital End Points Relevant to Autism and Co-Occurring Conditions: Protocol for a Multisite Observational Study
Source: JMIR Res Protoc. 2025 Oct 3;14:e71145. doi: 10.2196/71145 (PMC12534762; doi:10.2196/71145)
Supplement: Multimedia Appendix 2 [file resprot_v14i1e71145_app2.pdf]

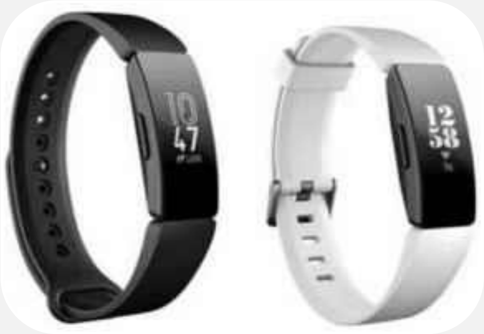

LEAP WAVE 3
MOBILE MEASURES MONTH

Participant Handbook

[V3: 22/11/2022]

CONTENTS

Page Number for:

Background	4
What does this study involve?	5
Overview	6
What data will be collected?	8
Fitbit	9
RADAR Active App	10
My Journ-E app	11
RADAR Passive App	12
What will happen to my data?	18
Privacy Policy	19
Instructions	21
My Journ-E app	22
RADAR Active App	24
RADAR Passive App	26
Fitbit	29
Contact and Support	32

Return to contents page

MEET THE TEAM

Hello and welcome to the Mobile Measures Month! We are the research team. We will be the key members who will help set you up on the wearable device and smartphone apps. We are also here to contact throughout the month for any support you need, to troubleshoot and to answer any queries or concerns you may have.

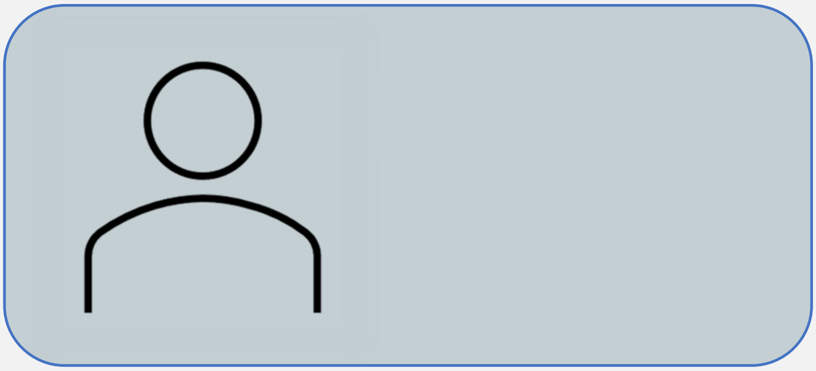

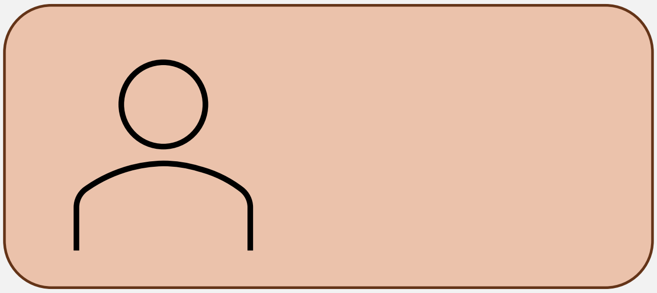

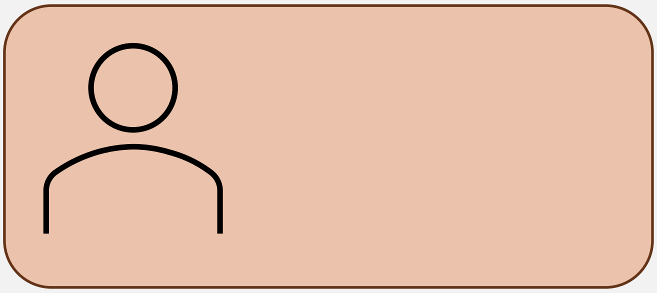

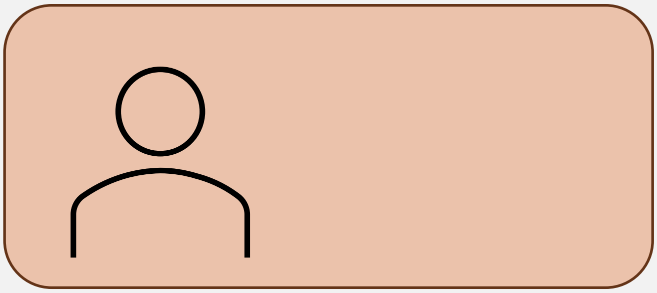

Return to contents page

BACKGROUND

The Mobile Measures Month is part of the Longitudinal European Autism Project, or LEAP study. You've been invited to participate because you have participated in the LEAP study before.

In previous waves of data collection, you have been invited to fill in questionnaires and come into the research centre to participate in various assessments.

We can't assume that information we collect during a study visit reflects how people usually think or feel, so in this new part of the study, we are interested in collecting data for a longer period during people's everyday lives. We are also interested in how objective data (e.g. data recorded by smartphones and a wearable device) relates to subjective data (how people report their thoughts and feelings).

For more information, please have a look at our Participant Information Sheet.

Return to contents page

WHAT IS INVOLVED?

There are 4 parts to this study.

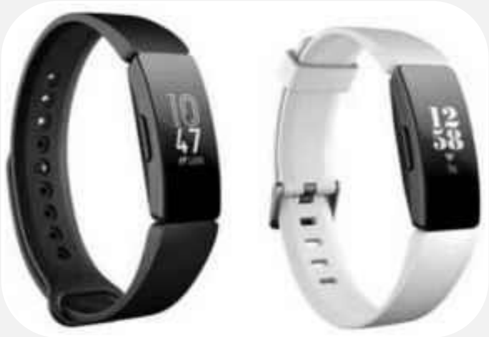

Fitbit

Wearing a Fitbit device

RADAR active app

Answering some questions on your smartphone

MyJournE app

Recording your mood on your smartphone

RADAR passive app

Installing an app that will record data without your input.

Return to contents page

OVERVIEW OF THE MONTH

We are inviting you to participate for one month (28 days). Here is what we will invite you to do during this time:		
**Set-up**	We will talk you through the study and setting up your devices via a video call lasting around 1 hour	
**Devices**	Wear a Fitbit for as much of the time as possible<br>Keep Fitbit and smartphone charged	
**Every day…**	You'll be notified to report on your **sleep** and **mood**. This will only take a few minutes each time.	
**Twice a week…**	You'll be notified to report on your **attention** levels. This will only take around a minute.	
**Twice in total…**	You'll be notified to report on your **social behaviour**. This will take around 5 minutes.	
**Feedback**	At the end of the month, you'll be notified to answer some questions on how you found it.<br>We'll also invite you to speak with us in more depth about your experience.	

Return to contents page

QUESTIONS SCHEDULE

Enrolment (set-up, practice questions)							
**App**	Day 1	Day 2	Day 3	Day 4	Day 5	Day 6	Day 7
							1
	Day 8	Day 9	Day 10	Day 11	Day 12	Day 13	Day 14
							2
	Day 15	Day 16	Day 17	Day 18	Day 19	Day 20	Day 21
							3
	Day 22	Day 23	Day 24	Day 25	Day 26	Day 27	Day 28
							4
**User Experience Interview**							

Mood logs

Sleep rating

Attention ratings

Social behaviour ratings

3 Week achieved

Return to contents page

WHAT DATA WILL BE COLLECTED?

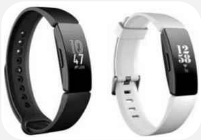

Return to contents page

FITBIT SMARTWATCH

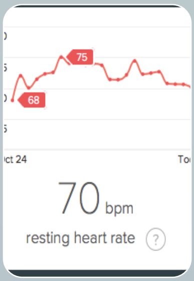

Heart rate & breathing

Fitbit detects your heart beats using a technique that uses light to measure blood flow. It also uses this to estimate breathing rate.

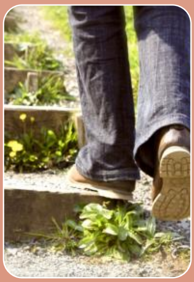

Activity levels

Using an accelerometer Fitbit works out your step count.

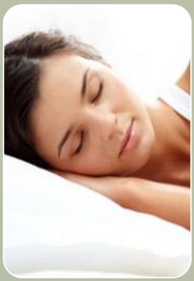

Sleep pattern

Using activity and heart rate data, Fitbit works out information about your sleep pattern.

Return to contents page

RADAR ACTIVE APP

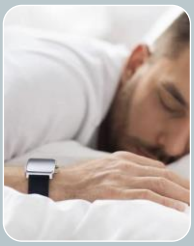

Sleep quality questions

Each morning you'll receive a notification to rate your previous night's sleep out of 100

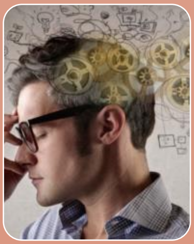

Attention questions

Twice a week, you'll receive a notification to answer four questions on your attention levels

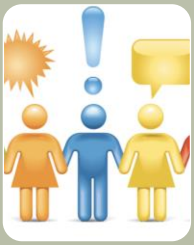

Social behaviour questions

Each fortnight you'll receive a notification to answer a questionnaire on your social and communication behaviour

Return to contents page

MY JOURN-E APP

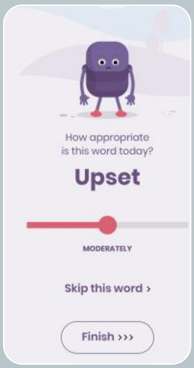

Context and mood log

You can choose to be reminded up to twice a day to log your mood and current context (where you are, what you're doing and who you're with).

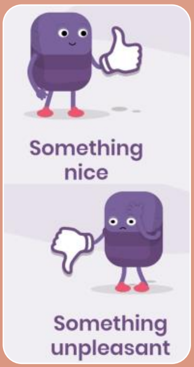

Event diary

You have the option to record nice or unpleasant events that may have affected your mood, whenever you like.

Return to contents page

RADAR PASSIVE APP

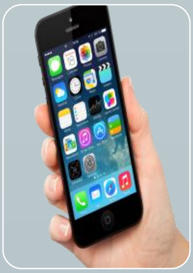

Smartphone use

We will collect data on how participants use their smartphones, for example, when and for how long people use social, entertainment, and productivity apps. Information about communication via phone calls and SMS will include duration/length and whether or not the communication was with a saved contact. No app, call or SMS content will be accessed.

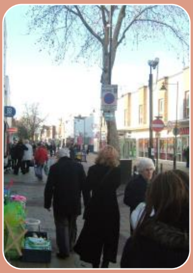

Environment

We will collect information from your phone about your environment. This includes how busy with people the environment is (indicated by how many Bluetooth devices are nearby). It will also include weather information and light levels. Light level data will contribute to information on sleep routines.

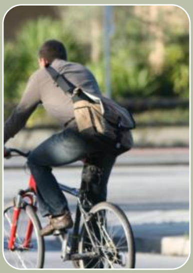

Activity and travel

Your smartphone's movement sensors (e.g. accelerometer) will contribute to our data on movement and activity levels. We will also collect data from your phone's Global Positioning System (GPS) and network connection. This data is automatically changed to hide participants' real locations.

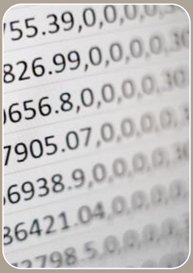

Other data

Data on battery levels, charging status, and switching on and off is collected in order to help analyse the other data.

Return to contents page

RADAR PASSIVE APP

More information on the data that is collected

Return to contents page

SMARTPHONE USE

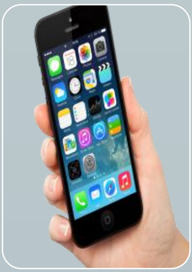

Apps

We will collect:

- App name
- When the app was opened, used or closed

We will not have access to any app content such as which websites have been visited.

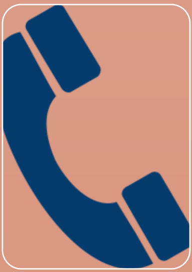

Call log

We will collect:

- Time of incoming/outgoing call
- Duration of call
- Whether the call was with a saved contact or not

We will not have access to call content

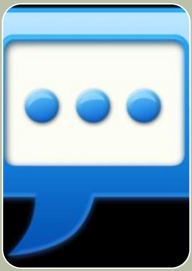

SMS log

We will collect:

- Time message sent/received
- Length of message
- Whether the message was to/from a saved contact

We will not have access to message content

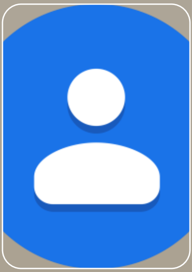

Contact list

The system will access this to record whether communication (phone calls and SMS) was with a saved contact or not. No phone numbers or contact details will be recorded. Additionally, we will collect:

- How many contacts are saved on the phone
- How many have been added or removed since last record

Return to contents page

ENVIRONMENT

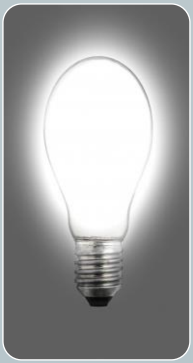

Light levels

This will help us examine sleep pattern.

We will collect:

- Light level (measured in lumens)

This is collected each time a change in light level is detected.

Bluetooth devices

Since most people own a smartphone with Bluetooth, number of devices gives an idea of how many people are nearby. For example, this may be quite low at home but higher in a supermarket or place of work.

- How many Bluetooth-enabled devices are nearby
- How many Bluetooth devices are paired to your phone

Return to contents page

ACTIVITY AND TRAVEL

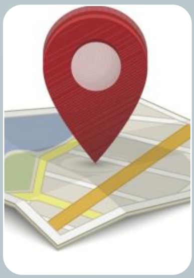

Relative location

Smartphones have onboard GPS that enables users to find their way around, e.g. using a maps app. Our system accesses your co-ordinates, but offsets these by a random number before recording them. This means we don't have access to a real location for you, but can still record information about travel.

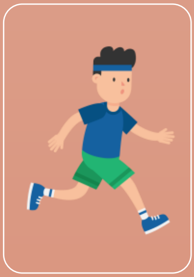

Activity sensors

We will collect data from your phone's onboard movement sensors. These are:

- Accelerometer
- Gyroscope
- Magnetometer

These provide additional information on travel and movement.

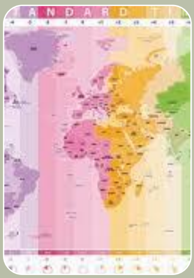

Time zone

For most people, this will not change during the Mobile Measures Month; however, we will record this if it does so that we can match up when data is recorded.

Return to contents page

OTHER SMARTPHONE DATA

Device information

We will collect

- Make and model of the smartphone
- Android version

Battery and power status

We will collect:

- Whether the phone battery is charging or discharging
- Time since the phone last switched on or restarted
- Lock and unlock events

Record counts and server status

We will record how much data is coming in and whether our connections are working. If we notice that data stops being recorded, we might contact you to see if we can help with any technical issues.

Return to contents page

HOW WILL WE ENSURE YOUR PRIVACY?

Return to contents page

DATA MANAGEMENT

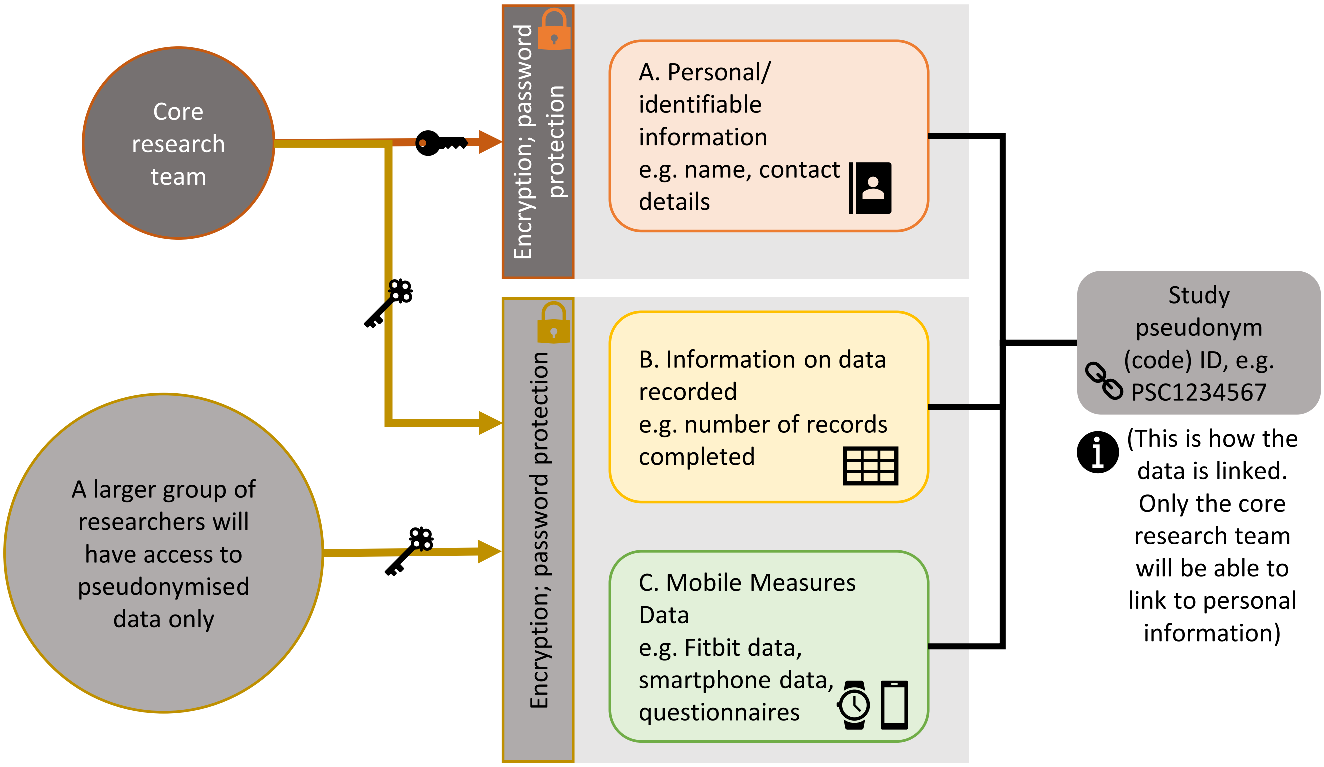

A: Your personal and contact information. This is kept separately to all other study data and will only be accessed by the core research team for the purpose of contacting you about the study.

B: Data recording information. The core research team will also access information about whether the mobile measures data is being successfully recorded. For example, whether the Fitbit device is syncing and whether app data is coming into the system. If we notice that data is not being recorded, e.g. due to a technical issue, we will contact you to resolve the issue.

C: The Mobile Measures data. This includes all the data coming in from the Fitbit and smartphone apps. This is kept separately to all information that could identify you. This data will only be accessed for analysis purposes, by named researchers who have applied to use the data for a specific project.

For more detail, please see our privacy policy.

Return to contents page

PRIVACY POLICY

We will use information from you for this research project, in the following ways:

Identifiable data

Any identifiable data such as names and addresses will be stored on a secure server within the university, specially designed for storing this type of information safely. All paper files will be kept in a locked filing cabinet. People will use this information to check your records to make sure the research is being done properly. The only people who will have access to information that identifies you will be people who need to contact you to check on your progress throughout the study or audit the data collection process. Name and contact information will not be used during data analysis and research publication; your contribution will be completely anonymous.

Protection and storage measures

To maintain your privacy, your name will be replaced with a code, and the information collected from the apps and Fitbit will only be associated with this code. It will also be encrypted (scrambled) so that only the research team can see it. Any information you provide us with via the apps or the Fitbit will not be checked or acted upon by members of the research team. Your de-identified data will be securely stored on servers managed by the Institute of Psychiatry, Psychology and Neuroscience, King's College London, and also on 3rd party servers (e.g. Fitbit), which can provide additional security and backup. This data will not be linked back to your personal information.

Sharing

Once initial quality control checks are done locally, all the de-identified Mobile Measures data will be sent to the AIMS-2-TRIALS common database (located in Paris, France) where a second secure identifying code will be assigned to you for additional data security. There, it will be put together with information collected at the other European institutions, and previous phases of this study. Storage of data collected as part of EU-AIMS is now funded by AIMS-2-TRIALS as a continuation of this previous project. All data is stored confidentially for a minimum of 15 years in accordance with European data protection laws. AIMS-2-TRIALS partners from all participating institutions will then be able to access the combined information.

When the time comes, will ask for your permission to audio record the feedback interview with you. With your permission, we may share those recordings with other EU-AIMS LEAP researchers, affiliated researchers at King's College London and selected external collaborators. The recordings will be transferred and stored on a secure server within the university designed to store personal data. Video/audio materials will never be shared with third parties without your explicit permission. We will not record your assessment unless you give explicit consent for us to do so.

Sharing of scientific data among researchers helps to speed the rate of scientific progress enormously. We will therefore ask you whether you are willing for your data to be shared in an anonymised fashion with other research groups or databases. Your data will be shared with other researchers around the world and used in future research projects after ethics approval. These projects can take place in universities, hospitals, non-profit groups, companies, and/or government laboratories. All researchers must respect the laws and ethical guidelines for biomedical research. To do that we will create an additional secure identifier, which will enable data collected within our project to be used safely and efficiently by other researchers outside of our project. This additional secure identifier will also be useful if you participate in other projects in the future.

When we go through the consent form with you, you can tell us if you do or do not wish to share your data beyond this project. We will then flag whether your data can be further shared. This decision will not affect your participation in any way.

Return to contents page

INSTRUCTIONS

We recommend going through these with a researcher during your enrolment call

MY JOURN-E

Set-up

We will take you through setting up the app on your smartphone during our enrolment video call. You will be asked to download the app from this link during the call.

How to use it?

You can use the app to record your context and mood at any time. Ideally, we'd like you to report on your mood twice per day. You will be able to set times for up to two reminders per day (e.g. midday and evening) to fill in the mood log. However, you can easily skip mood items or entire reports if it's not convenient.

Troubleshooting

If you have any questions or concerns, please contact the research team for more assistance.

MY JOURN-E SET UP

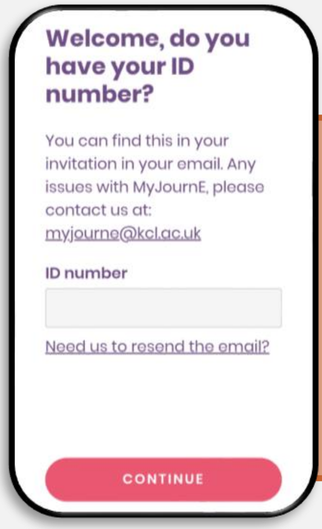

To sign-up, we will provide your participant ID number. You will be prompted to change your nickname which, can be edited later, on your account.

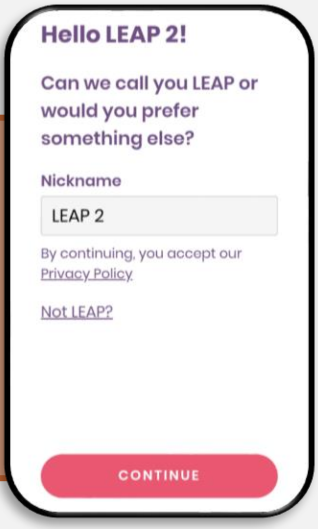

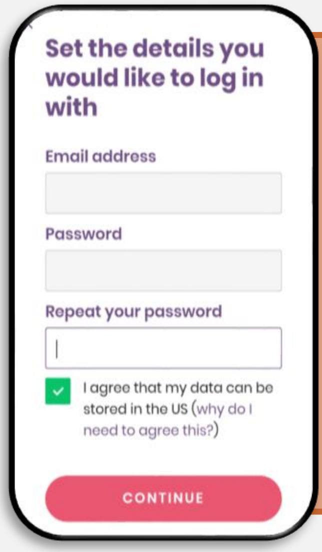

While you choose your own password, the email address will be automatically filled with an email we've allocated to you for the app. Once set-up you'll be asked about notification settings which are changeable in settings.

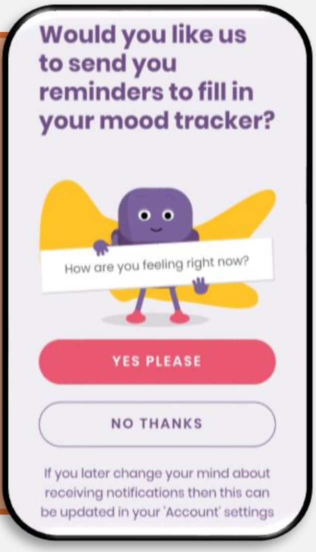

Return to contents page

RADAR ACTIVE APP

Set-up

We will take you through setting up the app on your smartphone during our enrolment video call. You will be asked to download the app from this link during the call.

How to use it?

The app will notify you when input is needed. If you select the notification, it will ask you to answer a question or short series of questions. You can answer by choosing the response that you feel fits best for you. You can also choose to skip any questions that you'd prefer not to answer.

Troubleshooting

If you have any questions or concerns, please contact the research team for more assistance.

RADAR ACTIVE APP SET UP

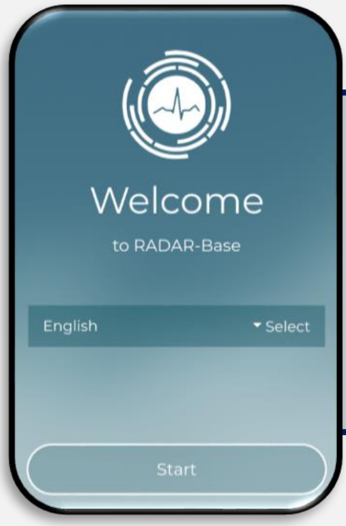

On downloading the app, you can select your preferred language. To enroll you in the study we will either present you a QR code or provide you a token to enter.

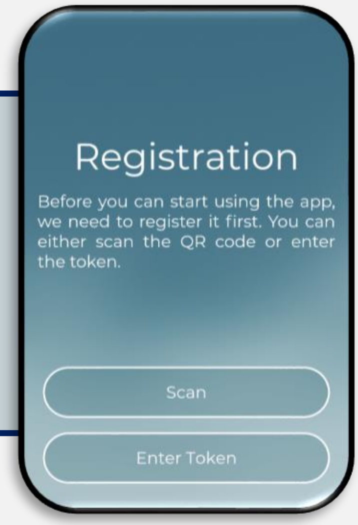

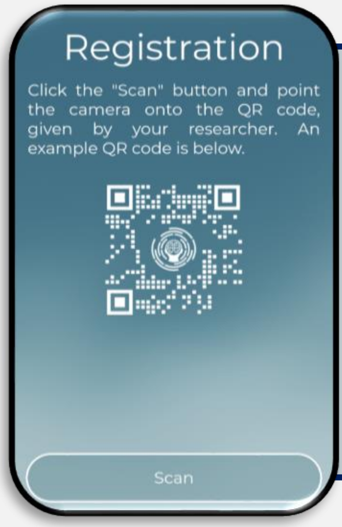

Once the QR code has been scanned or the token has been entered you'll be successfully registered as part of the study. This same registration process will be completed again for the passive app.

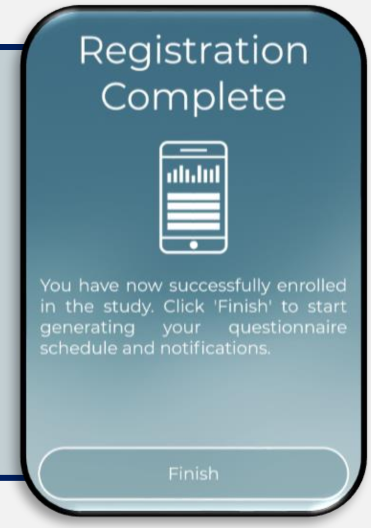

Return to contents page

RADAR PASSIVE APP

Set-up

We will take you through setting up the app on your smartphone during our enrolment video call. During the call, you will be asked to click this link to download the app. Note that some smartphone models have difficulty with this link. If that's the case for you, a researcher will support you to download the app.

How to use it?

The passive app doesn't need any input from you and should just run in the background.

Troubleshooting

If you have any questions or concerns, please contact the research team for more assistance.

RADAR PASSIVE APP SET UP

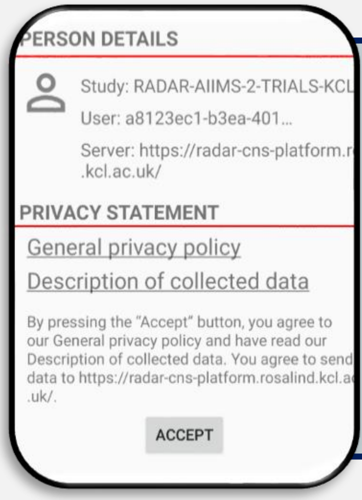

After the initial set-up, the app will present person details and privacy statements. Any questions can be asked during the enrolment call. Once you're happy you may accept or withdraw participation.

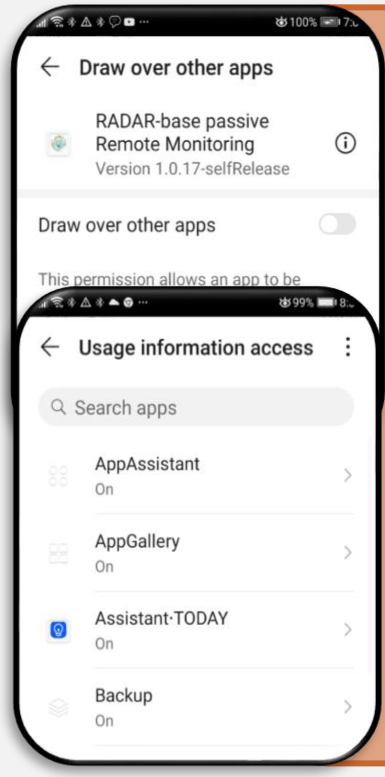

Allow the app to draw over other apps and press back.

A pop-up will appear for enabling usage tracking which keeps track of the apps you use. By pressing OK it will show a list of the apps you can allow or disallow access to.

When you're satisfied with the app access permissions, the back arrow will take you back to the passive app.

RADAR PASSIVE APP SET UP

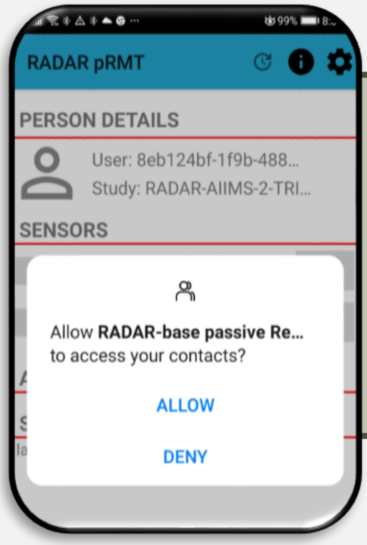

Allow the app to drop over other apps. Additional pop-ups will appear asking for permission to collect data previously detailed in the handbook. Allow the app all permissions for which you feel comfortable giving.

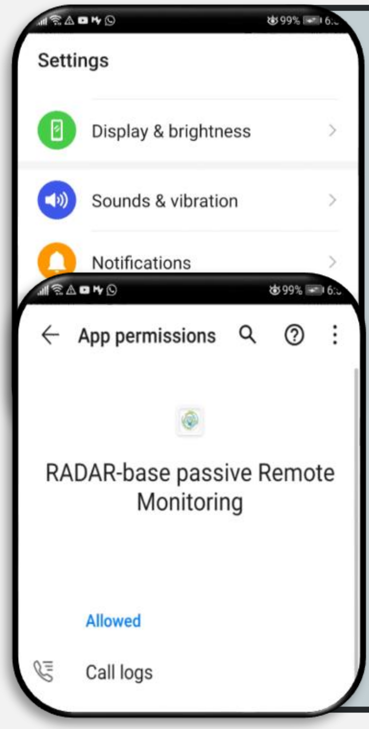

For any reason, if you change your mind about the permissions, you can change them by going to settings.

By going to ‘Apps’, finding the ‘Passive App’ and going to Permissions you can find a list of the data you previously allowed or denied the app.

Simply select whichever data you want to edit permissions for and allow or deny access for the app.

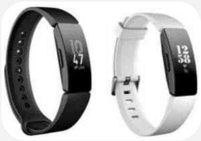

Return to contents page

FITBIT

Set-up

We will take you through setting up your Fitbit and pairing it with your smartphone during our enrolment video call. We will also show you how to care for and charge the device.

How to wear it?

Fitbit is a widely-used device that is worn like a watch. You will have the opportunity to choose a strap which you find suitable. To allow us to collect data as accurately as possible, the strap should be worn securely so that the back of your device is in contact with your skin. The device should be positioned one finger width above your wrist bone. It should feel snug, but not so tight that it is uncomfortable or can't move slightly.

Troubleshooting

Fitbit provide webpages that can be helpful if you have minor difficulties. However, please do not hesitate to contact the research team for more assistance.

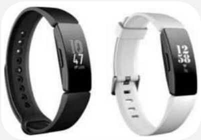

Return to contents page

FITBIT SET UP

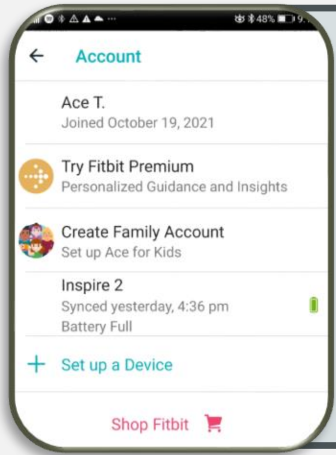

Please download the Fitbit app. Your log-in details will be provided during your enrolment appointment. Go to account to update personal information then tap 'Set up a Device' to connect your Fitbit to your phone.

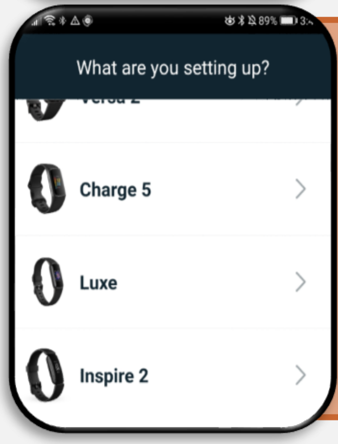

From the list of Fitbit devices that appear select Inspire 2 and tap 'set up'. The Privacy Policy will appear, and you may accept after your happy with the information stated. The app will show how to charge the device.

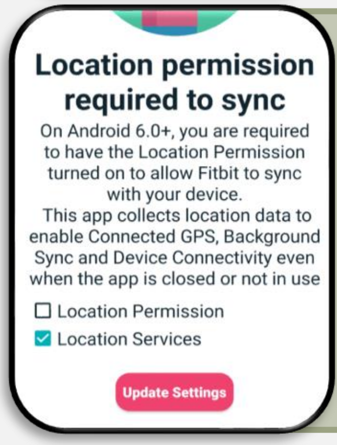

If you have an Android 6.0+, 'Location Permission' is required for the app and Fitbit to sync via Bluetooth. Fitbit does not record or track your location. Allow the permission if you are happy to, and update settings. You can then exit the screen.

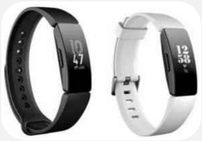

Return to contents page

FITBIT SET UP

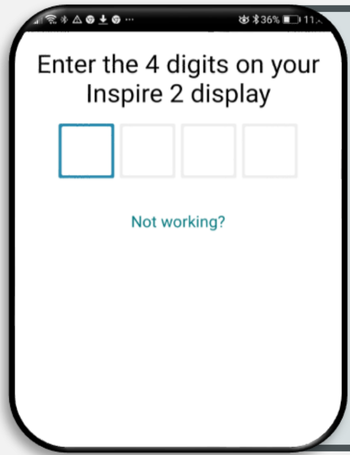

Your phone will automatically begin looking for your Fitbit device. Once it detects the device, the Fitbit will display a 4-digit number to type into your phone. A tick will appear on the Fitbit to confirm the devices are synced.

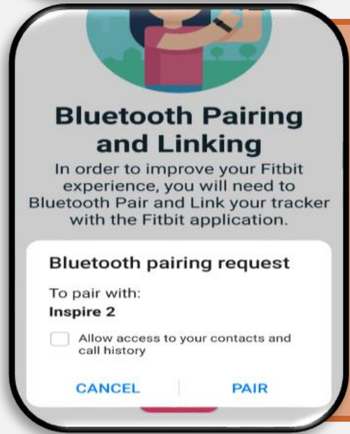

A Bluetooth pairing request will appear. You may choose to allow access to your contacts and call history. One last pop-up will ask to link Fitbit with Inspire 2.

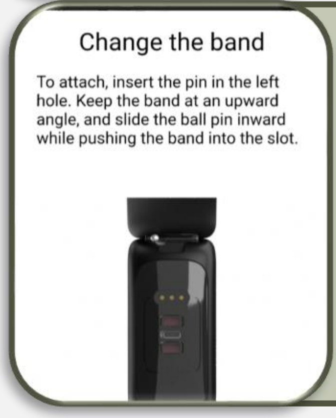

The app will display how to change the Fitbit band; swiping on the Fitbit; locking the Fitbit screen and showing a summary of how to wear the Fitbit safely. You do not need to download the TileTM app or the premium version of the Fitbit app to participate in our study.

Return to contents page

CONTACT AND SUPPORT

If you have a concern about any aspect of this study, please speak to the researchers who will do their best to answer your question.

Contact Information: Dr Isabel Yorke (Izzy)

Phone: 02078485369

Email: isabel.d.yorke@kcl.ac.uk

If they are unable to answer your questions or you have a complaint, please contact the principal investigator, Professor Emily Simonoff, Emily.Simonoff@kcl.ac.uk.

You are also welcome to seek further advice from Patient Advice and Liaison Service under phone number 020 3299 3601.

If you feel that you would benefit from further support with your mental health, please visit:

https://www.nhs.uk/conditions/stress-anxiety-depression/mental-health-helplines/

Thank you for reading this document and for considering taking part in this study.
